# The m^6^A reader MhYTP2 regulates *MdMLO19* mRNA stability and antioxidant genes translation efficiency conferring powdery mildew resistance in apple

**DOI:** 10.1111/pbi.13733

**Published:** 2021-11-17

**Authors:** Tianli Guo, Changhai Liu, Fanxin Meng, Liu Hu, Xiaomin Fu, Zehua Yang, Na Wang, Qi Jiang, Xiuzhi Zhang, Fengwang Ma

**Affiliations:** ^1^ State Key Laboratory of Crop Stress Biology for Arid Areas/Shaanxi Key Laboratory of Apple College of Horticulture Northwest A&F University Yangling Shaanxi China

**Keywords:** YTH domain, m^6^A reader, mRNA stability, translation efficiency, powdery mildew, apple

## Abstract

N^6^‐methyladenosine (m^6^A) reader protein plays an important role in trichome morphology, developmental timing and morphogenesis in *Arabidopsis*. However, the function of m^6^A readers in plant‐microbe interaction remains unclear. Here, a *Malus* YTH‐domain family protein MhYTP2 was initially characterized as an m^6^A reader. *MhYTP2* overexpression increased mRNA m^6^A modification level and translation efficiency. The m^6^A in the exon regions appeared to destabilize the mRNAs, whereas m^6^A in the untranslated regions positively correlated with the associated mRNA abundance. *MhYTP2* overexpression enhanced apple powdery mildew resistance, possibly by rapidly degrading the bound mRNAs of *MdMLO19* and *MdMLO19‐X1* and improving the translation efficiency of the antioxidant genes. To conclude, the results shed light on the apple m^6^A profile, the effect of MhYTP2 on m^6^A profile, and the m^6^A roles in *MdMLO19* and *MdMLO19‐X1* mRNAs stability and *glutamate dehydrogenase 1‐like MdGDH1L* mRNA translation efficiency.

## Introduction

Biotic stresses, including fungal and bacterial diseases, inevitably challenge plant survival and reproduction. Powdery mildew (PM) caused by *Podosphaera leucotricha* is a devastating disease of apple (*Malus × domestica* Borkh.), one of the most widely cultivated and economically important fruits worldwide. PM causes white spots on young green tissues, and the infected leaves crinkle, curl and prematurely drop. Blossoms and fruits are not the primary targets of the PM fungi; however, infection of these tissues is also possible (Foulongne *et al*., 2003; Turechek and Carroll, 2004; Xiao *et al*., 2001). Subsequently, PM affects apple fruit development and quality, reducing fruit yield and market value.

Plants have evolved several defence mechanisms over time. The plant disease stress responses involve molecular, physiological and cellular adaptations. At the molecular level, plants control gene expression levels by reprogramming their genetic machinery, via which they implement defence mechanisms and increase stress tolerance to minimize the effects of the accompanying biological damage (Fujita *et al*., 2006). Genetic regulatory networks, including post‐transcriptional control of gene expression, serve as powerful strategies in disease stress responses (Huh and Paek, 2014). For example, apple *Mildew Locus O 19* (*MdMLO19*) plays a pivotal role in PM resistance; its knockdown significantly induced PM resistance (Pessina *et al*., 2014, 2016). Meanwhile, the absence of *Mlo* primes the responsiveness for the onset of multiple defence functions in barley (Büschges *et al*., 1997; Wolter *et al*., 1993). RNA binding proteins (RBPs), the important components of such regulatory networks that remain active during and after transcription (Chen and Varani, 2013), identify and combine with target RNAs via the RNA binding domains (RBDs). Among the various post‐transcriptional modifications, N^6^‐methyladenosine (m^6^A) RNA methylation is one of the most pivotal internal modifications and a conserved post‐transcriptional mechanism that enriches and regulates genetic information in eukaryotes (Yue *et al*., 2019). The m^6^A modification accounts for 80% of the RNA base modifications in eukaryotic cells (Kierzek and Kierzek, 2003). Studies have shown that m^6^A modification in plants influences various growth and development phenomena, including embryo development and seed germination (Vespa *et al*., 2004; Zhong *et al*., 2008), apical meristem and flower development (Bodi *et al*., 2012; Duan *et al*., 2017; Růžička *et al*., 2017; Shen *et al*., 2016), microspore development (Zhang *et al*., 2019), root development (Chen *et al*., 2018), leaf surface trichome development (Arribas‐Hernández *et al*., 2018; Bodi *et al*., 2012; Chen *et al*., 2018; Scutenaire *et al*., 2018; Vespa *et al*., 2004; Wei *et al*., 2018), and fruit ripening (Zhou and Tian, 2019).

Plant m^6^A modification influences stress responses also. Studies have shown that the *Arabidopsis* m^6^A reader proteins evolutionarily conserved C‐terminal region1 (ECT1) and evolutionarily conserved C‐terminal region2 (ECT2) interact with stress response protein calcineurin B‐like‐interacting protein kinase1 (CIPK1) (Ok *et al*., 2005), and ECT2 affects mRNA relocation under heat stress (Scutenaire *et al*., 2018; Wei *et al*., 2018). *Arabidopsis* m^6^A demethylase ALKBH9B interacts with the viral coat protein and RNA and modulates alfalfa mosaic viral infection (Martínez‐Pérez *et al*., 2017). Meanwhile, the m^6^A hypomethylation plays a positive role in drought response in maize (Miao *et al*., 2020). Studies have demonstrated that these functions of m^6^A modification in stress responses were achieved through m^6^A regulating mRNA processing and metabolism, including mRNA transport, degradation (Luo *et al*., 2014; Meyer and Jaffrey, 2014; Schwartz *et al*., 2013; Shi and Wei, 2019), stability (Wang *et al*., 2014), splicing (Zhao *et al*., 2014) and translation efficiency (Wang *et al*., 2015; Zhou *et al*., 2015). The role of methylation depends on the m^6^A reader protein (Bi *et al*., 2019; Shi *et al*., 2019). *Arabidopsis* ECT2 protein, containing an YTH domain, has been proven to be an m^6^A reader protein (Scutenaire *et al*., 2018; Wei *et al*., 2018). However, the role of apple RBP MhYTP2, a homologue of *Arabidopsis* ECT2, in the m^6^A binding function is unknown. Moreover, the m^6^A methylation machinery and the m^6^A characteristics and functions responsible for regulating pathological processes of horticultural crops remain largely unknown.

One of the major stress response mechanisms at the physiological level is the increase in the levels of antioxidants, such as ascorbic acid (AsA) and glutathione (GSH), to reduce stress‐induced intracellular ROS accumulation (Gururani and Venkatesh, 2015). The AsA‐GSH cycle, an antioxidant system, plays an important role in scavenging hydrogen peroxide (H_2_O_2_) under stress (Wang *et al*., 2012). GSH, a major endogenous antioxidant, participates in H_2_O_2_ detoxification via various glutathione peroxidases (Gu and Chauhan, 2015). Meanwhile, the GSH synthesis regulated by GDH helps plants absorb the excessive ${\text{NH}}_{4}^{+}$ to produce glutamate, for the first committed step in GSH synthesis (Skopelitis *et al*., 2006; Tercé‐Laforgue *et al*., 2013; Yan *et al*., 2021). However, the direct role of GDH in PM resistance in apple is unclear.

Therefore, the present study aimed at characterizing the *Malus* YTH domain‐containing RNA binding protein 2 (MhYTP2). The *MhYTP2* was cloned from *Malus hupehensis* (pamp.) Rehd., a PM‐resistant genotype, and overexpressed in *Malus domestica* cv. ‘Roya Gala’ to explore its function in PM resistance. Further, a global mRNA methylation and transcription analysis of the *35S::MhYTP2* line OE‐2 and the WT plants was performed to examine the influence of *MhYTP2* on the overall methylation level. RNA immunoprecipitation (RIP)‐sequencing was conducted to identify the targets of MhYTP2, and ribosome profiling (Ribo‐seq) was performed to analyse the role of *MhYTP2* in regulating translation efficiency. Collectively, our work demonstrates that the m^6^A binding function of MhYTP2 controls PM resistance by binding and affecting the mRNA stability of *MdMLO19* and *MdMLO19‐X1* while facilitating the translation efficiency of antioxidant genes.

## Results

### Overexpression of *MhYTP2* increases resistance to PM

**Figure 1:**
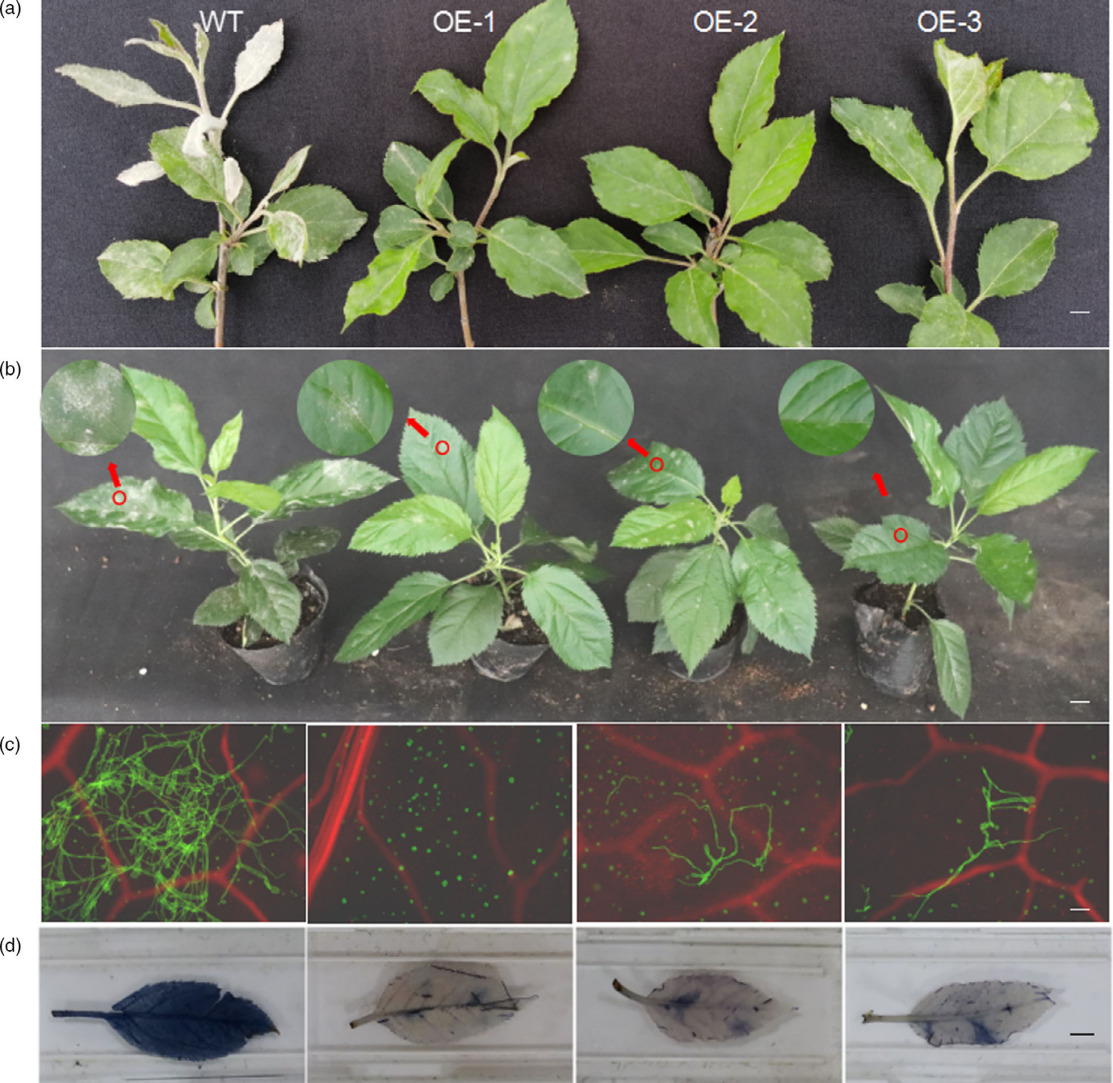
Overexpression of *MhYTP2* increases resistance to powdery mildew (PM) under field and laboratory conditions. (a) Disease severity with PM in the *35S::MhYTP2* lines and the wild type (WT) plants under natural conditions. Bar, 1 cm. (b) Disease severity recorded 15 days after inoculation with PM in the *35S::MhYTP2* lines and the WT plants under laboratory conditions. Bar, 1 cm. (c) Spread of the pathogenic hyphae in the inoculated leaves under laboratory conditions. Bar, 1 mm. (d) Trypan blue staining of the PM inoculated leaves under laboratory conditions. Bar, 1 cm.

Three *35S::MhYTP*2 lines (OE‐1, OE‐2, OE‐3) showed increased resistance to PM compared with the WT plants in the pots under field conditions (Figure 1a). The *35S::MhYTP2* lines and the WT plants were also tested for their susceptibility to PM under laboratory conditions by artificial inoculation in the following study. Similarly, the *35S::MhYTP2* lines demonstrated lower disease severity than the WT plants 15 days after PM inoculation under laboratory conditions. Although the leaves of the *35S::MhYTP2* lines were partially infected, the spread of spores on the adaxial leaf surface was significantly reduced compared with the WT (Figure 1b). The WGA staining revealed that the pathogen’s spread in the *35S::MhYTP2* lines was significantly reduced compared with the WT (Figure 1c). Besides, the trypan blue staining indicated fewer disease symptoms in *35S::MhYTP2* lines than in the WT (Figure 1d).

### Overexpression of *MhYTP2* modifies the expression levels of m^6^A writers and erasers

**Figure 2:**
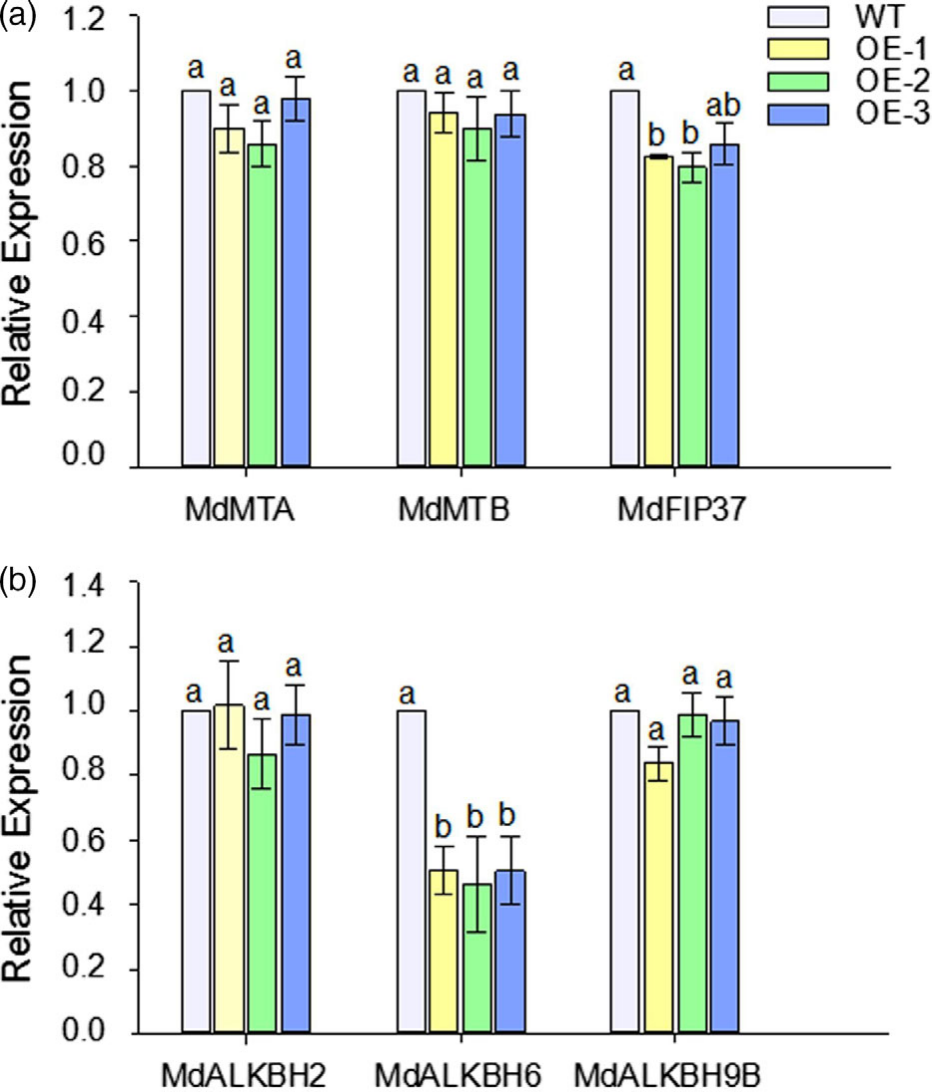
Overexpression of *MhYTP2* alters the transcript levels of methyltransferase and demethylase genes. The transcript levels of the (a) methyltransferases *MdMTA*, *MdMTB* and *MdFIP37* and (b) the demethylases *MdALKBH2*, *MdALKBH6* and *MdALKBH9B* in the *35S::MhYTP2* lines and the WT plants. Quantitative RT‐PCR was performed to determine the transcript levels using the *MdActin* gene as the internal control. Data are presented as mean ± standard deviation (*n* = 4). Different letters indicate significant differences between WT and *35S::MhYTP2* plants, based on one‐way ANOVA and Tukey’s multiple range test (*P* < 0.05).

*Arabidopsis* ECT2 protein has been reported as an m^6^A reader (Scutenaire *et al*., 2018; Wei *et al*., 2018). Sequence alignment revealed that MhYTP2 is a homolog of *Arabidopsis* ECT2 protein with a YTH domain (Figure S1). Therefore, we hypothesized that MhYTP2 is also an m^6^A reader and may affect the mRNA methylation level. The expression levels of several important methyltransferase and demethylase genes (Wei *et al*., 2018) in the *35S::MhYTP2* lines and WT plants were analysed to investigate the effect of *MhYTP2* on the mRNA methylation levels. The quantitative real‐time PCR (RT‐PCR) analysis revealed that the expression levels of the N^6^‐adenosine‐methyltransferase genes *MT‐A70‐like* (*MdMTA*) and *non‐catalytic subunit MTB* (*MdMTB*) in the *35S::MhYTP2* lines were slightly lower than that in the WT plants. Meanwhile, the *FKBP12‐interacting protein of 37* *kDa* (*MdFIP37*) in the *35S::MhYTP2* lines was significantly lower than that in the WT plants (Figure 2a). The transcript levels of demethylases *MdALKBH2* and *MdALKBH9B* were similar in the *35S::MhYTP2* lines and WT plants; however, the transcript level of *MdALKBH6* was significantly lower in the *35S::MhYTP2* lines than in the WT (Figure 2b). These observations indicate that MhYTP2 may influence the overall methylation level in apple.

### Variations in methylome between *35S::MhYTP2* line OE‐2 and WT

**Figure 3:**
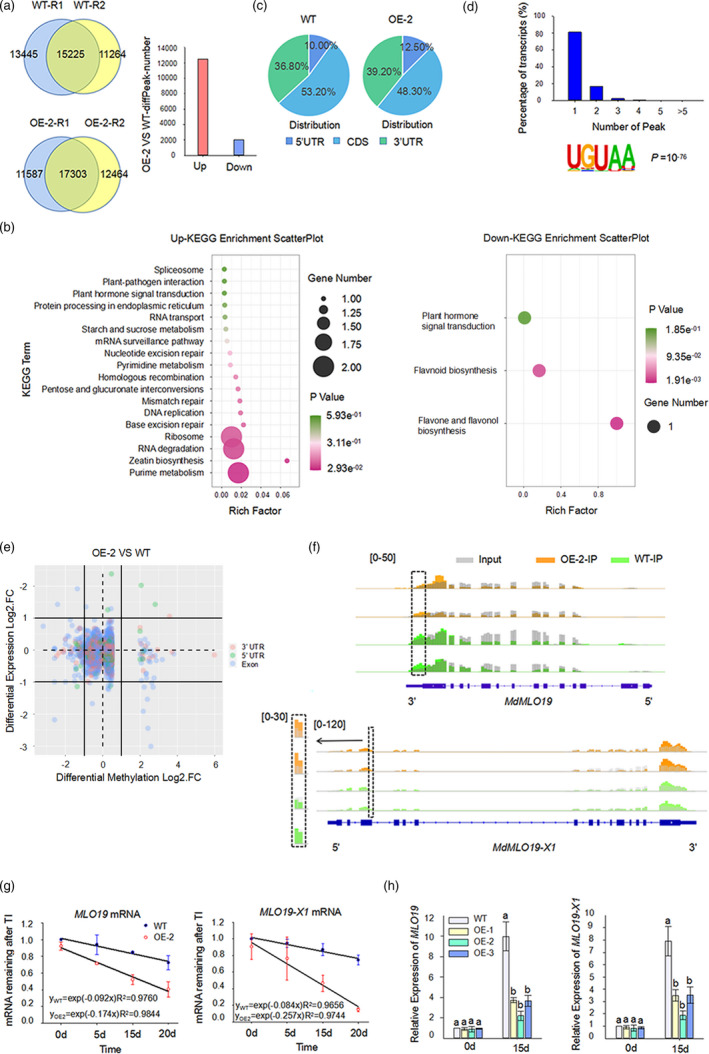
Variations in methylome between *35S::MhYTP2* line OE‐2 and WT. (a) Venn diagrams showing the overlap of m^6^A peaks identified in two independent m^6^A‐seq experiments from the leaves of WT and *35S::MhYTP2* line OE‐2. Only the peaks identified in two biological replicates were the confident peaks and used for subsequent data analysis. The number of different m^6^A‐modified transcripts in the *35S::MhYTP2* line OE‐2 and the WT. (b) KEGG analysis of the m^6^A‐containing transcripts identified via m^6^A‐seq. KEGG analysis was performed using the upregulated and downregulated transcripts. (c) Metagenomic profiles of peak summit distributions along with the transcripts composed of three rescaled non‐overlapping segments (5′ UTR, CDS, and 3′ UTR; UTR, untranslated region; CDS, coding sequence). (d) Proportion of the m^6^A‐modified transcripts containing different m^6^A peaks and binding motifs identified by HOMER based on the identified MhYTP2‐binding peaks. (e) Diagram showing the correlation between m^6^A mRNA methylation and gene transcript levels in apple. (f) m^6^A modification levels of *MdMLO19* and *MdMLO19‐X1* in the *35S::MhYTP2* line OE‐2 and the WT plants. The arrows indicate the gene direction from 5′ to 3′ end. (g) Lifetime of *MdMLO19* and *MdMLO19‐X1* mRNAs in the *35S::MhYTP2* line OE‐2 and the WT plants treated with actinomycin D in the transcription inhibition assay. Data are represented as mean ± standard deviation (SD, *n* = 3 biological replicates * 3 technical replicates). TI, transcription inhibition. (h) *MdMLO19* and *MdMLO19‐X1* expression levels in the *35S::MhYTP2* lines and the WT plants. Data are represented as mean ± standard deviation (SD, *n* = 3). Different letters indicate significant differences between WT and *35S::MhYTP2* plants on the same day, based on one‐way ANOVA and Tukey’s multiple range test (*P* < 0.05).

Further, m^6^A‐seq (Dominissini *et al*., 2013) was performed to assess the transcriptome‐wide m^6^A methylation in the WT and *35S::MhYTP2* line OE‐2 using leaf samples to investigate the potential influence of MhYTP2 on mRNA methylation. The mRNA samples from WT and *35S::MhYTP2* line OE‐2 were fragmented into approximately 100 nucleotide‐long oligonucleotides (input) before IP using an anti‐m^6^A affinity purified antibody. Libraries were prepared from input control and IP fragments and subjected to parallel sequencing. The mRNA samples were prepared to perform two independent m^6^A‐seq experiments. High‐confidence m^6^A peaks detected in both biological replicates for each line were used for subsequent analysis. A total of 15,225 and 17,303 high‐confidence m^6^A peaks were identified from the WT and *35S::MhYTP2* line OE‐2 leaves (fold change ≥ 2; *P* < 0.05), respectively. Here, 12,484 transcripts displayed increased m^6^A levels, while only 2024 transcripts showed decreased m^6^A enrichment in *35S::MhYTP2* line OE‐2 compared with the WT plants (fold change ≥ 2; *P* < 0.05), suggesting a global increase in m^6^A methylation in *35S::MhYTP2* line OE‐2 compared with the WT plants (Figure 3a) and the influence of MhYTP2 on the overall methylation level. Further, Kyoto Encyclopedia of Genes and Genomes (KEGG) enrichment analysis of m^6^A‐enriched transcripts differently between *35S::MhYTP2* line OE‐2 and WT was performed to gain functional insights into the role of *MhYTP2*. The analysis showed that the upregulated transcripts in *35S::MhYTP2* line OE‐2 were enriched in RNA degradation and plant‐pathogen interaction pathways. Downregulated transcripts were mainly enriched in the hormone signal transduction and biosynthesis of flavonoid pathways, implicating the crucial role of *MhYTP2* in multiple biological processes (Figure 3b). Further, the distribution of m^6^A peaks in the whole apple transcriptome was analysed. Each transcript was divided into three non‐overlapping segments based on the reference annotation: 5′ untranslated region (UTR), coding sequence (CDS) and 3′ UTR. As shown in Figure 3c, the meta‐genomic profiles of m^6^A peaks in all samples indicated that m^6^A modifications were highly enriched around the CDSs and the 3′ UTRs. Further, the distributions of m^6^A peaks in the WT and *35S::MhYTP2* line OE‐2 plants were analysed. The percentage of m^6^A peaks in the CDS regions was lower in the *35S::MhYTP2* line OE‐2 plants (48.30%) than that in the WT plants (53.20%), while those in the 5′ UTRs (12.50% vs. 10.00%) and the 3′ UTRs (39.20% vs. 36.80%) were higher. The m^6^A modifications around the UTRs in the *35S::MhYTP2* line OE‐2 were highly enriched than in the WT plants (Figure 3c), revealing the effect of MhYTP2 on the stability and translation of the m^6^A‐modified mRNA (Luo *et al*., 2020; Wang *et al*., 2014).

Among the gene transcripts containing m^6^A modification, the majority contained one m^6^A peak (81.04%), followed by a few with two m^6^A peaks (16.27%); only a few exhibited three or more peaks (2.69%) (Figure 3d), with levels consistent with those in *Arabidopsis* and tomato (Luo *et al*., 2014; Wan *et al*., 2015; Zhou *et al*., 2019). Besides, hypergeometric optimization of motif enrichment (HOMER; http://homer.ucsd.edu/homer/) was applied (Heinz *et al*., 2010) to identify the sequence motifs enriched within the m^6^A peaks in apple. Clustering of the m^6^A peaks using HOMER identified the “URUAY” sequence motif in apple (Figure 3d), similar to that in *Arabidopsis* (Wei *et al*., 2018), tomato (Zhou *et al*., 2019), and maize (Miao *et al*., 2020). These data suggest the conservation in m^6^A modification across plant species.

Studies have reported that m^6^A deposition influences mRNA abundance (Duan *et al*., 2017; Luo *et al*., 2014; Shen *et al*., 2016; Wan *et al*., 2015; Wang *et al*., 2014; Wei *et al*., 2018; Zhao and Roundtree, 2017). Therefore, RNA‐seq was performed to evaluate the potential correlation between m^6^A mRNA methylation and gene transcript levels in apple. Comparison of the differentially expressed genes (DEGs; fold change ≥ 2; *P* < 0.05) identified via RNA‐seq with the transcripts showing altered m^6^A levels between the *35S::MhYTP2* line OE‐2 and WT plants revealed that the m^6^A modification in the exon regions appeared to destabilize the mRNAs, whereas m^6^A in the UTRs was positively correlated with the abundance of associated mRNAs (Figure 3e). *MdMLO19*, a well‐known S‐gene involved in apple defence against PM (Pessina *et al*., 2014, 2016), was not identified in the *35S::MhYTP2* line OE‐2; however, the 3′ UTR of *MdMLO19* was m^6^A‐modified in the WT plants. In addition, *MdMLO19‐X1* was identified in the *35S::MhYTP2* line OE‐2 with m^6^A hypermethylation in the exon regions than the WT plants (Figure 3f). *MdMLO19‐X1* is an isoform of the previously reported *MdMLO19* (Pessina *et al*., 2014, 2016), with an mRNA sequence similarity of 52.96%, and an amino acid sequence similarity of 63.01% (Table S1). In fact, in addition to *MdMLO19* and *MdMLO19‐X1*, *MdMLO1*, *MdMLO1‐like* and *MLO11‐like X2* that have not been tested for their function in PM resistance in apple were also found with variations in methylome between *35S::MhYTP2* line OE‐2 and WT.

Furthermore, m^6^A methylation has been demonstrated to decrease mRNA stability (Duan *et al*., 2017; Shen *et al*., 2016; Wang *et al*., 2014; Zhao *et al*., 2017). Considering the role of m^6^A modification in exon regions in mRNA destabilization and that in UTRs in mRNA stability, we hypothesized that MhYTP2 promoted the degradation of *MdMLO19* and *MdMLO19‐X1* transcripts. Therefore, the lifetime of *MdMLO19* and *MdMLO19‐X1* transcripts was measured by blocking the transcription with actinomycin D. The *MdMLO19* and *MdMLO19‐X1* transcripts were degraded rapidly after actinomycin D treatment in the *35S::MhYTP2* line OE‐2 compared with the WT plants (Figure 3g), which suggests that m^6^A modification promotes *MdMLO19* and *MdMLO19‐X1* mRNAs degradation. These observations indicate that the site‐specific m^6^A modification destabilizes *MdMLO19* and *MdMLO19‐X1* mRNAs. Further quantitative RT‐PCR analysis revealed significantly lower expression levels of *MdMLO19* and *MdMLO19‐X1* in the *35S::MhYTP2* line OE‐2 than the WT plants after PM inoculation (Figure 3h).

### YTH‐domain protein MhYTP2 is an m^6^A reader and MhYTP2 associates with m^6^A‐containing *MdMLO19* and *MdMLO19‐X1*

**Figure 4:**
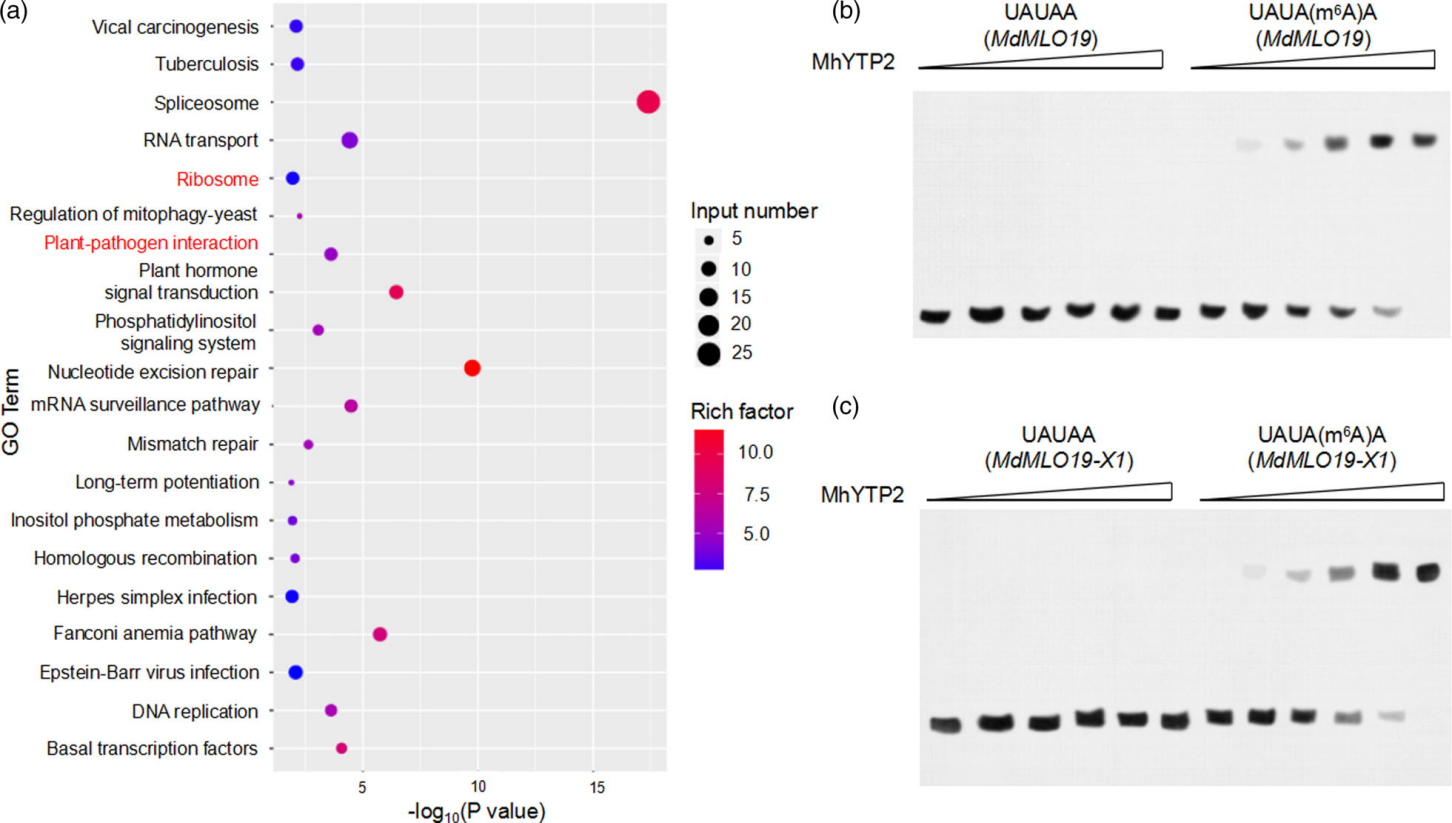
Target genes of MhYTP2 identified by RIP‐seq and EMSA. (a) GO analysis of the MhYTP2‐binding protein transcripts identified in RIP‐seq (*n* = 3). EMSA validation of the interaction between (b) MhYTP2 and m^6^A‐modified *MdMLO19* mRNA and (c) MhYTP2 and m^6^A‐modified *MdMLO19‐X1* mRNA.

MhYTP2 was identified as a homolog of *Arabidopsis* ECT2 protein with a YTH domain (Figure S1). Studies have proven ECT2 as an m^6^A reader protein (Scutenaire *et al*., 2018; Wei *et al*., 2018). RIP sequencing was performed using *35S::MhYTP2‐Flag* apple calli line MhYTP2‐F2 to determine whether MhYTP2 can bind with m^6^A‐containing *MdMLO19* and *MdMLO19‐X1* mRNAs (Figure S2). Unfortunately, *MdMLO19* and *MdMLO19‐X1* mRNAs were not detected in MhYTP2‐flag IP mRNA samples (Figure 4a). Consequently, the MhYTP2‐His protein from *Escherichia coli* BL21 was expressed and purified to perform the *in vitro* RNA electrophoretic mobility shift assay (EMSA) with synthetic 42‐mer RNAs containing either m^6^A or A. The EMSA showed a direct binding between MhYTP2 and the m^6^A‐modified *MdMLO19* and *MdMLO19‐X1* mRNAs, with fourteen and eight “URUAY” motifs in their mRNA sequences, respectively (Appendix S1). (Figure 4b). The result indicated that MhYTP2 binds to RNA transcripts that harbour m^6^A sites. Thus, MhYTP2, like human and *Arabidopsis* YTH‐domain family proteins, was confirmed as an m^6^A reader protein. Therefore, the study’s observations indicate that MhYTP2 binding with and degrading *MdMLO19* and *MdMLO19‐X1* mRNAs may be a possible route via which MhYTP2 improved apple resistance to PM.

### Overexpression of *MhYTP2* promotes ribosome occupancy

**Figure 5:**
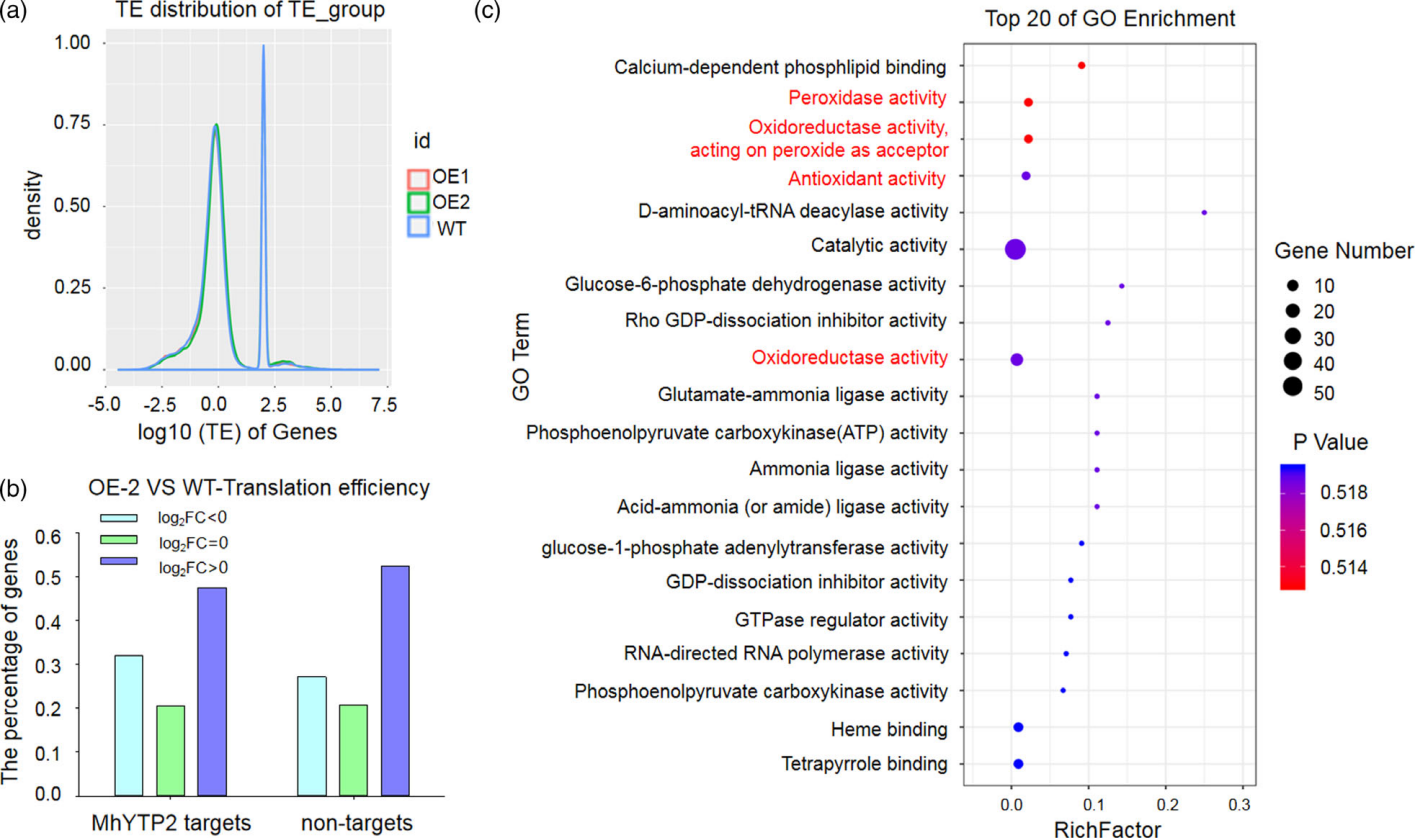
MhYTP2 enhances the translation of MhYTP2 target and non‐target transcripts. (a) Overexpression of *MhYTP2* enhanced translation efficiency. Density distribution of the log10‐fold changes in translation efficiency between the *35S::MhYTP2* line OE‐2 and the WT plants. (b) Overexpression of *MhYTP2* enhanced the translation efficiency of MhYTP2 targets and non‐targets. (c) GO analysis of the transcripts with different translation efficiencies identified in Ribo‐seq.

Gene ontology (GO) analysis of the RIP‐binding transcripts showed that the bound transcripts in *35S::MhYTP2* line OE‐2 compared with the WT plants were enriched for plant‐pathogen interaction and ribosome (Figure 4a). Recent studies showed that m^6^A modification regulates translation efficiency (Wang *et al*., 2015; Zhou *et al*., 2021). Therefore, ribosome profiling was used to assess the ribosome density of each transcript mRNA with or without *MhYTP2* overexpression to explore the role of MhYTP2 in mRNA translation. Overexpression of *MhYTP2* changed the main distribution range of the translation efficiency data set (Figure 5a). Further analysis based on the abundance ratio of mRNA in the polysomal RNA versus the total RNA (ribosome occupancy) (Merchante *et al*., 2015) combined with RIP‐seq results revealed that *MhYTP2* overexpression enhanced the translation efficiency of MhYTP2 targets and non‐targets (Figure 5b). GO analysis of the different ribosome occupancy transcripts (fold change ≥ 2; *P* < 0.05) showed that the transcripts were enriched in the antioxidation system (Figure 5c), suggesting the effect of MhYTP2 on the apple antioxidant system.

### Overexpression of *MhYTP2* enhances *MdGDH1L* translation efficiency

**Figure 6:**
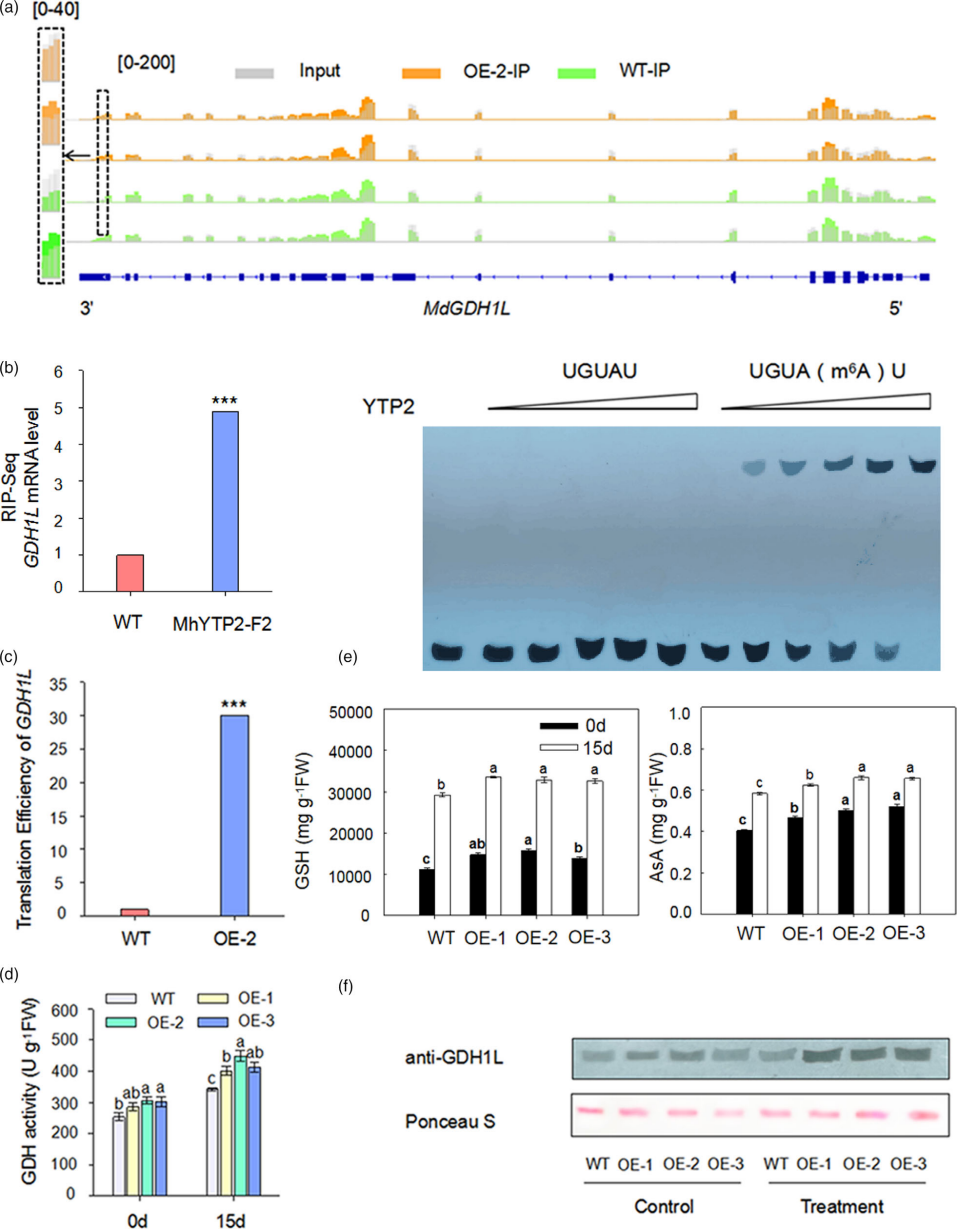
MhYTP2 promotes the translation efficiency of *MdGDH1L*. (a) m^6^A modification levels of *MdGDH1L* in the *35S::* *MhYTP2* line OE‐2 and the WT plants. The arrows indicate the gene direction from the 5′ to 3′ end. (b) RIP‐seq and EMSA validation of the interaction between MhYTP2 and m^6^A‐modified *MdGDH1L* mRNA. MhYTP2‐F2 represents transgenic *35S::MhYTP2‐Flag* apple callus line 2. (c) Translation efficiency of *MdGDH1L* in the *35S::MhYTP2* line OE‐2 and the WT plants. (d) Changes in GDH enzyme activities in apple leaves under normal and infected conditions. (e) Changes in GSH and AsA accumulation. Parameters were measured at the start and 15 days after inoculating PM. Data are represented as mean ± standard deviation (SD, *n* = 3). Different letters indicate significant differences between WT and *35S::MhYTP2* plants on the same day, based on one‐way ANOVA and Tukey’s multiple range test (*P* < 0.05) in (d) and (e). FW, fresh weight. (f) MdGDH1L protein expression in the *35S::MhYTP2* lines and the WT plants at the start and 15 days after inoculating PM. Asterisks indicate significant differences (***P < 0.001).

The different ribosome occupancy transcripts in *35S::MhYTP2* line OE‐2 compared with the WT plants were enriched in the antioxidation enzymes (Figure 5c), including MdGDH1L, which affects the synthesis of GSH. The m^6^A‐seq analysis of the *35S::MhYTP2* line OE‐2 and the WT plants showed that the *MdGDH1L* mRNA contains m^6^A sites at 3′UTR. The m^6^A modification level of *MdGDH1L* was significantly higher in *35S::MhYTP2* line OE‐2 than that in WT plants (Figure 6a). The RNA immunoprecipitation‐seq conducted using *35S:MhYTP2‐Flag* apple calli line MhYTP2‐F2 (Figure S2) showed that *M* *dGDH1L* mRNA was enriched in MhYTP2‐flag IP mRNA samples (Figure 6b). Therefore, MhYTP2‐His protein from BL21 *Escherichia coli* was expressed and purified to perform the *in vitro* RNA EMSA with synthetic 42‐mer RNAs containing either m^6^A or A to gain insight into the *MdGDH1L* m^6^A binding activity of MhYTP2. The EMSA showed that MhYTP2 bound with m^6^A‐modified *MdGDH1L* mRNA (Figure 6b). The *MdGDH1L* mRNA sequence contains seven “URUAY” motifs (Appendix S1). Furthermore, an overall increase in translation efficiency of the *MdGDH1L* mRNA was observed by ribosome profiling in *35S::MhYTP2* line OE‐2 (Figure 6c). Meanwhile, the activity of the GDH was significantly higher in *35S::MhYTP2* lines than in the WT (Figure 6d). Further evaluation revealed that GSH and AsA levels in *35S::MhYTP2* lines were higher than the WT plants under normal and PM inoculated conditions (Figure 6e). These findings indicate that *MhYTP2* overexpression improved its target *MdGDH1L* mRNA translation efficiency leading to more MdGDH1L protein and elevated antioxidant activity, resulting in enhanced PM resistance (Figure 6f). These data suggest that critical gene in the antioxidation system undergo m^6^A‐mediated post‐transcriptional regulation, facilitating translation to increase protein content in response to stress.

### MhYTP2 modulates genes encoding transcription elongation factors and translation initiation factor

**Figure 7:**
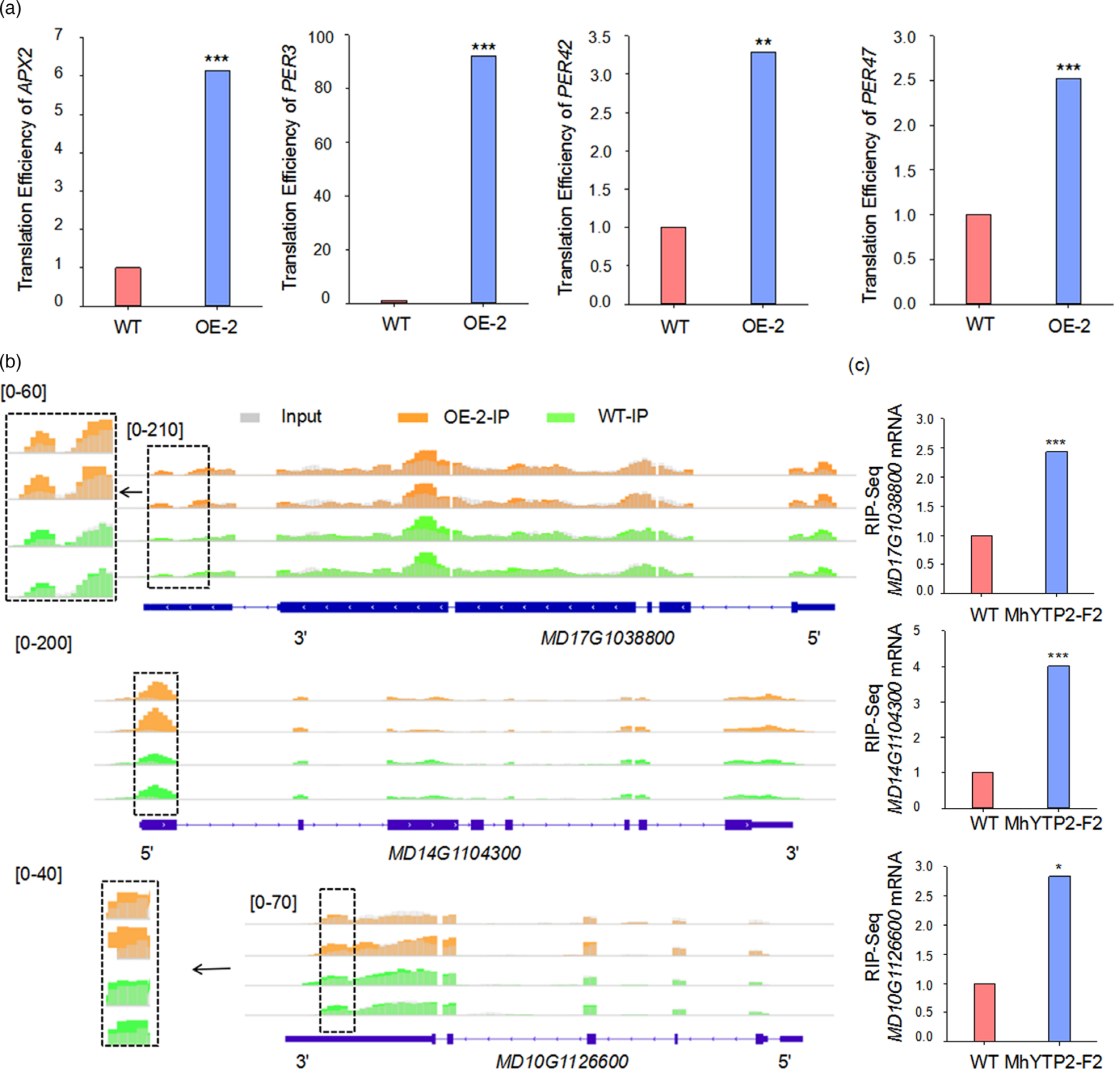
MhYTP2 modulates genes encoding transcription elongation factors and translation initiation factor and indirectly enhances translation efficiency of several antioxidant genes. (a) Translation efficiency of *MdAPX2*, *MdPER3*, *MdPER42*, and *MdPER47* in the *35S::MhYTP2* line OE‐2 and the WT plants. (b) m^6^A modification levels of *MD17G1038800*, *MD14G1104300* and *MD10G1126600* in the *35S::MhYTP2* line OE‐2 and the WT plants. The arrows indicate the gene direction from the 5' to 3' end. (c) RIP‐seq validation of the MhYTP2‐binding m^6^A‐modified *MD17G1038800*, *MD14G1104300*, and *MD10G1126600* mRNAs. MhYTP2‐F2 represents transgenic *35S::MhYTP2‐Flag* apple callus line 2. Asterisks indicate significant differences (***P < 0.001; **P < 0.01; *P < 0.05).

Further, the effect of MhYTP2 on the translation efficiency of other genes involved in the antioxidation system was evaluated based on the abundance ratio of mRNA in the polysomal RNA versus the total RNA (Merchante *et al*., 2015). The genes *L‐ascorbate peroxidase* (*APX*) and *peroxidase* (*PER*) exhibited significant changes in translation efficiency when *MhYTP2* was overexpressed (Figure 7a). Besides, the activities of APX and POD were significantly higher in *35S::MhYTP2* lines than in the WT (Figure S3). This change could not be due to m^6^A deposition because the transcripts of these genes were not m^6^A‐modified according to the m^6^A‐seq data sets. Therefore, we speculated that MhYTP2 might regulate the translation efficiency of numerous transcripts beyond direct m^6^A modification. As expected, *MhYTP2* overexpression altered the translation efficiency of mRNAs of several antioxidation‐related genes, such as *APX2*, *PER3*, *PER42* and *PER47*, which do not contain m^6^A modification.

**Figure 8:**
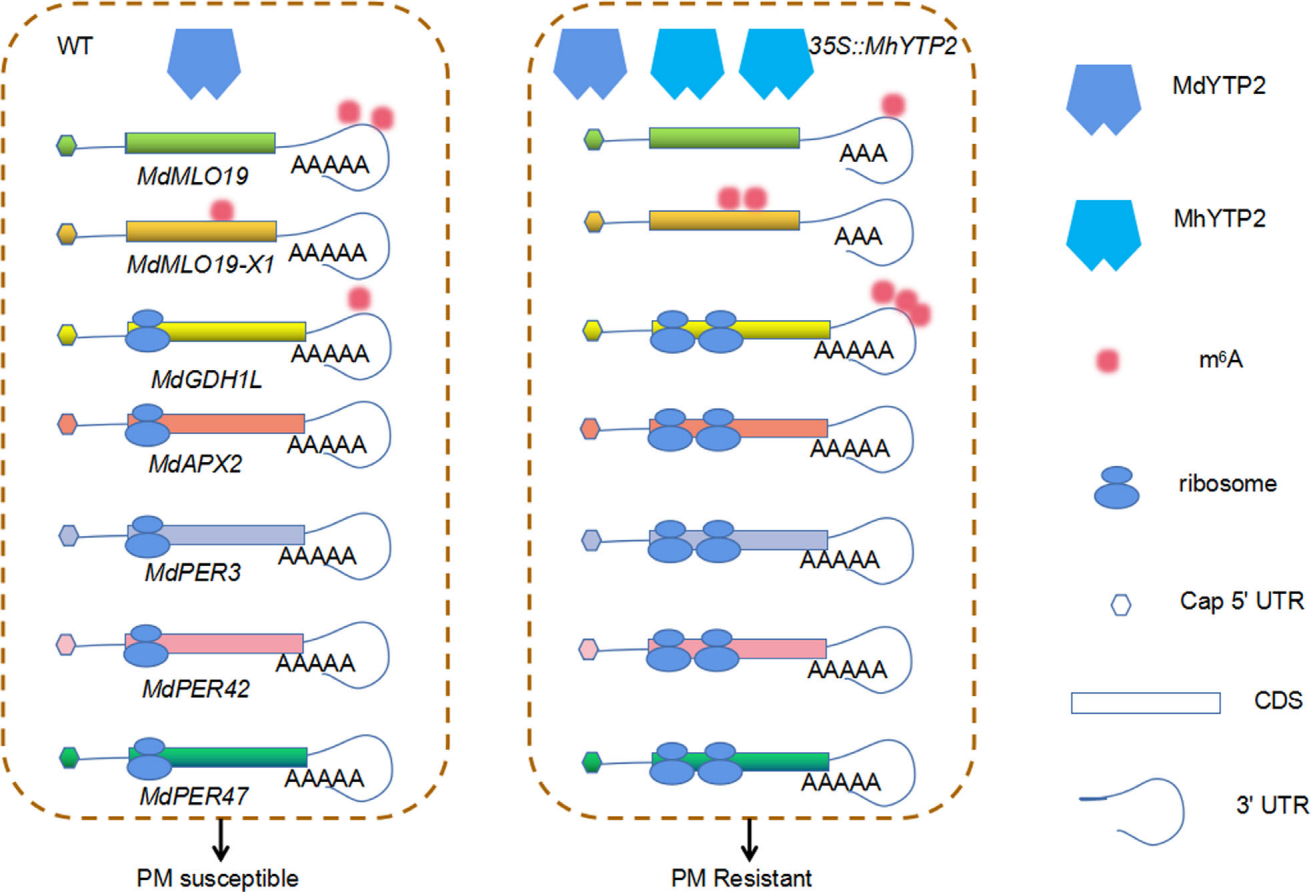
m^6^A reader protein MhYTP2 regulates apple resistance to PM.

Detailed analysis of the m^6^A‐seq and RIP‐seq data revealed that the transcripts of genes encoding transcription elongation factors (*MD17G1038800* and *MD14G1104300*) and translation initiation factor (*MD10G1126600*) (Table S2), which play pivotal roles in facilitating protein synthesis by promoting the elongation of mRNA transcription and initiation of mRNA translation, respectively, exhibited m^6^A hypermethylation in the UTRs in *35S::MhYTP2* line OE‐2 (Figure 7b). RIP analysis showed direct interactions between MhYTP2 and the transcripts of *MD17G1038800*, *MD14G1104300* and *MD10G1126600* (Figure 7c). Taken together, MhYTP2 may modulate the translation efficiency of its non‐targets indirectly by regulating m^6^A‐modified mRNAs of transcription elongation factors and translation initiation factor in addition to modulation of translation efficiency via direct binding with the m^6^A‐modified target, such as *MdGDH1L* mRNA. The results collectively indicate that MhYTP2 increased PM resistance by rapidly degrading *MdMLO19* and *MdMLO19‐X1* mRNAs and increasing the antioxidant genes translation efficiency. Based on these results, a model has been proposed for the MhYTP2 function in apple PM disease resistance (Figure 8).

## Discussion

Several researchers have investigated m^6^A of mammalians, and the discovery of m^6^A demethylases and the mapping of the m^6^A methylomes in the mammalian systems have indicated that m^6^A methylation of mRNA is a reversible and dynamic process with regulatory functions (Dominissini *et al*., 2012; Fu *et al*., 2014; Jia and Fu, 2013; Jia *et al*., 2011; Meyer *et al*., 2012; Nilsen, 2014; Zheng *et al*., 2013). Further characterization of the mammalian reader protein YTHDF2 that recognizes m^6^A modifications and subsequently affects mRNA stability indicated the importance of m^6^A in post‐transcriptional regulation of gene expression (Fu *et al*., 2014). Studies have shown that YTHDF1 mediates translation and promotes translation efficiency (Wang *et al*., 2015) and YTHDF3 plays a significant role in modulating the translation of m^6^A‐modified mRNAs (Li *et al*., 2017). Although the data available is scarce, the situation in plants seems to be similar. The *Arabidopsis* YTH‐domain m^6^A reader protein ECT2 accelerated the degradation of three transcripts related to trichome morphogenesis, thereby affecting trichome branching (Scutenaire *et al*., 2018; Wei *et al*., 2018). Meanwhile, the present study focused on the m^6^A binding properties of MhYTP2 and its effect on mRNA and confirmed that the apple YTH‐domain protein MhYTP2 is an m^6^A reader.

Overexpression of *MhYTP2* in apple modified the expression levels of genes encoding m^6^A writers and erasers. The m^6^A writers with methyltransferase function for *MdFIP37*and that with demethylase function for *MdALKBH6* were significantly downregulated in the *35S::MhYTP2* lines. Based on the finding that *MhYTP2* modified the expression levels of genes encoding m^6^A writers and erasers, the transcriptome‐wide m^6^A sequencing was conducted. Transcriptome‐wide m^6^A distributions in the *35S::MhYTP2* line OE‐2 and WT apple plants indicated that the methylation sites are highly conserved compared with that in *Arabidopsis* and tomato (Scutenaire *et al*., 2018; Wei *et al*., 2018; Zhou *et al*., 2019), indicating a fundamental function of m^6^A in plants.

Further, the relationship between m^6^A distribution and gene expression was analysed. Importantly, the discovered features of the m^6^A distribution associated with gene expression in apple mRNAs were found different from *Arabidopsis*. The distinct enrichment of m^6^A in the exon regions was correlated with the overall upregulation of mRNA expression level in *Arabidopsis* (Scutenaire *et al*., 2018; Wei *et al*., 2018), while that in the apple was correlated with the overall downregulation of mRNA expression level in this study. Noticeably, m^6^A methylation at UTRs was positively correlated with gene expression. These observations indicated that the correlation between methylation level and gene expression depends on the modification site. Moreover, the m^6^A modifications in the *35S::MhYTP2* line OE‐2 plants were highly enriched around the UTRs than that in the WT plants, which reveals that MhYTP2 may mainly affect the stability and translation of the m^6^A‐modified mRNA (Anderson *et al*., 2018; Meyer *et al*., 2015). We speculate that the role MhYTP2 plays on the stability and translation of the m^6^A‐modified mRNAs associated with disease resistance may affect apple defence against PM.

Studies have shown that the natural and artificial loss‐of‐function mutations of *MLO* S‐genes reduce susceptibility to PM pathogens (Büschges *et al*., 1997). *MdMLO19* induced by PM is a functional S‐gene in apple, and its knockdown substantially reduced PM susceptibility (Pessina *et al*., 2016). Nevertheless, the regulatory mechanisms underlying the *MdMLO19* signalling pathway, especially the regulation of the *MdMLO19* gene at the post‐transcriptional level, remain largely unknown. In this work, *MdMLO19* and *MdMLO19‐X1*, an isoform of *MdMLO19*, underwent m^6^A‐mediated post‐transcriptional regulation, which showed significant decrease in m^6^A modification levels of *MdMLO19* in the 3′ UTR and significant induction in m^6^A modification levels of *MdMLO19‐X1* in the exon regions, respectively, in the *35S::MhYTP2* line OE‐2 compared with the WT plants. The changed m^6^A modification levels of *MdMLO19* and *MdMLO19‐X1* mRNAs accelerated their degradation in *35S::MhYTP2* line OE‐2. These findings provide a novel information that *MdMLO19* and *MdMLO19‐X1* mRNAs are post‐transcriptionally regulated via m^6^A‐modification.

The m^6^A modification has also been demonstrated to affect translation efficiency beyond mRNA stability (Wang *et al*., 2015). The *35S::MhYTP2* line OE‐2 of this study showed a global increase in m^6^A modification levels (Figure 3a) and translation efficiency (Figure 5b). Upregulation of *GDH* genes has been observed under stress conditions (Fontaine *et al*., 2012; Lehmann and Skrok, 2010). In this study, the m^6^A modification at 3′ UTR and translation efficiency of MhYTP2‐binding *MdGDH1L* were significantly higher in the *35S::MhYTP2* line OE‐2 than in the WT plants (Figures 6a, c). Further, the changes in translation efficiency appeared to be directly regulated by m^6^A deposition on the transcripts of *MdGDH1L* or indirectly by m^6^A‐mediated regulation of transcription elongation factors and translation initiation factor for *MdAPX2*, *MdPER3*, *MdPER42*, and *MdPER47*. The improved translation efficiency of *MdGDH1L* made the transgenic plants resistant to PM. These findings collectively reveal a novel layer of gene regulation in the antioxidation system signalling pathway and establish a link between the m^6^A‐mediated antioxidation system and apple disease resistance.

The present study identifying the function of the apple m^6^A reader MhYTP2 in mRNA stability and translation sheds light on the mechanisms through which m^6^A functions in RNA metabolism and plant disease resistance. Considering the multiple roles of MhYTP2 on m^6^A metabolism, the regulation identified may have an essential function in many biological processes.

## Methods

### WT and *35S::MhYTP2* plant treatment

The WT plants and the *35S::MhYTP2* plants of the *Malus domestica* cv. ‘Roya Gala’ were used in this study (Liu *et al*., 2019). The WT and *35S::MhYTP2* plants generated by tissue culture were initially grown on MS agar containing 0.2 mg/L 6‐benzylaminopurine (6‐BA) and 0.2 mg/L indoleacetic acid (IAA) at 23 °C, 60 µmol/m^2^/s, and 14 h photoperiod. After 45 days of growth on MS agar, the plantlets were transferred to the rooting MS agar media containing 0.5 mg/L indole butyric acid and 0.5 mg/L IAA and maintained for another 45 days. The WT and *35S::MhYTP2* plantlets with similar size were transferred to small plastic pots (8.5 cm × 8.5 cm × 7.5 cm) containing a mixture of soil and perlite (1 : 1, v : v). After 60 days of growth in a growth chamber under 50% relative humidity, 28 °C, and a long photoperiod (16 h:8 h, light:dark), plants of similar size were selected to test the response to PM inoculation.

The inoculation experiments following the dry‐brushing of healthy leaves with *P. leucotricha* obtained from the diseased leaves were carried out in a growth chamber at 50% relative humidity and 28 °C in the dark for 24 h. Disease severity on all inoculated leaves was visually assessed 15 days after the inoculation. Six leaves per plant were harvested before and after inoculation and immediately frozen in liquid nitrogen. All frozen samples were stored at −80 °C. The phenotype recording and trypan blue and leaf fungal hyphae WGA staining were carried out 15 days after inoculation. In addition, the disease severity under field conditions was determined manually based on the size of white spots on leaves.

### Microscopy

WGA‐AF 488 (Molecular Probes, Karlsruhe, Germany) was used to stain the fungal hyphae, and propidium iodide (Sigma, Shanghai, China) to stain the plant cells. Samples were infiltrated with a staining solution (1 µg/L propidium iodide, 10 µg/L WGA‐AF 488; 0.02% Tween 20 in PBS pH 7.4), incubated for 30 min, and washed in 1× PBS (137 mm NaCl, 2.7 mmKCl, 12 mm phosphate buffer, pH 7.4). Images were recorded on a TCS‐SP5 confocal microscope (Leica, Bensheim, Germany), at an excitation wavelength of 488 nm and a detection wavelength of 500–540 nm. The mCherry fluorescence (an excitation wavelength of 561 nm and a detection wavelength of 580–630 nm) was used for live‐cell imaging of fungal hyphae in apple leaves. Green‐fluorescence protein was excited with a 488 nm laser, and the emission was detected at 495–530 nm for fungal hyphae. The samples were observed, and images were recorded using a CoolSNAP‐HQ charge‐coupled device camera (Photometrics, Shanghai, China) controlled with the imaging software MetaMorph (Universal Imaging, Los Angeles, CA, USA).

### GDH activity assay and antioxidant metabolite analysis

GDH activity and the GSH and AsA content of apple leaves were determined using the specific kits (Sangon Biotech, Shanghai, China), following the manufacturer’s instructions.

### Ribosome profiling

Ribo‐seq using apple callus was performed at lcsciences (LC‐Bio Corporation, Hangzhou, China; https://www.lcsciences.com/), according to the protocol described by Zhou *et al*. (2018).

### RNA extraction and quantification and gene expression analysis

Total RNA was extracted from the collected samples using a spin column plant total RNA purification kit (Sangon Biotech), according to the manufacturer’s instructions. The cDNA was reverse transcribed from the total RNA using a PrimeScript ® RT reagent Kit with gDNA Eraser (Takara, Dalian, China). Further, quantitative RT‐PCR was conducted on a LightCycler® (Roche, Basel, Switzerland) 96 real‐time PCR detection system (Roche) using the ChamQ SYBR quantitative PCR Master Mix (Vazyme Biotech, Nanjing, China), according to the manufacturer’s instructions. The apple *MdActin* (XM_008344381) was used as the internal control gene. The relative expression level of the target gene transcript was determined by the $2^{‐\Delta \Delta C_{t}}$ method (Kim *et al*., 2009; Livak and Schmittgen, 2001). All primers are listed in Table S3.

### RIP‐seq and EMSA

RNA immunoprecipitation was performed as previously described by Zhou *et al*. (2019). The *35S::MhYTP2‐Flag* ‘Orin’ cultivar’s calli were flash‐frozen and crushed in liquid nitrogen, and the frozen powder was homogenized in 15.75 mL of lysis buffer (100 mm Tris‐HCl, pH 7.5, 150 mm NaCl, 0.5% IGEPAL, and 1% plant protease inhibitor cocktail from Sigma). The crude extract was clarified by centrifugation at 3000 g for 10 min at 4 °C. The immunoprecipitation was performed using 11.25 mL of the crude extract incubated for 1.5 h at 4 °C on a rotating wheel with 120 µL of Flag‐trap magnetic beads (Chromotek, Munich, Germany). RNA was eluted from the beads with 400 µL of guanidium extraction buffer and precipitated overnight at −22 °C with 800 µL of pure ethanol. RNA was precipitated by centrifugation, resuspended in 350 µL of RTL buffer (Qiagen, Hilden, Germany), purified according to the manufacturer’s instructions, and eluted in 15 µL of RNase‐free water. The RNA solution was concentrated to 6 µL using the RNA clean and concentrator kit (Zymo Research, Irvine, CA, USA. Code: R1015). Similarly, RNA was extracted from 200 µL of crude extract to monitor RNA in the input fraction. RNA samples from both the eluate and input fractions were treated with DNase as part of the Qiagen RNEasy purification procedure. The beads were resuspended in 15 µL of Laemmli buffer and incubated for 5 min at 95 °C, and the supernatant was collected from the beads using a magnetic device. Sequencing was done on an Illumina HiSeq machine with 2 × 100 cycles of Solexa paired‐end sequencing. The experiment was repeated three times. Whole RIP and protein immunoprecipitation experiments were conducted using the freshly prepared and harvested calli for each replicate.

Electrophoretic mobility shift assay was performed following a previously reported method (Wang *et al*., 2014; Wei *et al*., 2018) with minor modifications. The digoxin‐labelled RNA oligonucleotides used to assay the binding affinity of MhYTP2‐His are listed in Appendix S2. The RNA probe was used at 4 nmol concentration. The concentration of MhYTP2‐His ranged from 0 to 2000 nm.

### mRNA purification and m^6^A‐seq

Total RNA was extracted from the collected samples using a spin column plant total RNA purification kit (Sangon Biotech). Intact mRNA was purified from total RNA using Dynabeads mRNA purification kit (Ambion, Austin, TX, USA. Code: 61006).

The m^6^A IP was carried out following a previously reported procedure with slight modifications (Chen *et al*., 2015; Dominissini *et al*., 2013). The purified mRNA samples from the WT and *35S::MhYTP2* line OE‐2 plants were digested using DNase I to generate 100 nucleotide‐long fragments by incubating at 94 °C for 5 min in RNA fragmentation buffer (10 mm ZnCl2, 10 mm, Tris‐HCl, pH 7.0). The reaction was stopped using 0.05 m EDTA (Ambion Code: AM8740), followed by phenol‐chloroform extraction and ethanol precipitation. For the m^6^A‐seq, anti‐m^6^A polyclonal antibody (10 µg antibody for 5 µg mRNAs; Synaptic Systems (Chromotek. Code: 202003)) was incubated in IP buffer containing 150 mm NaCl, 0.1% NP‐40 (v/v), 10 mm Tris‐HCl (pH 7.4), and 300 U/mL RNase inhibitor (Promega, Madison, WI, USA. Code: N2112S) for 2 h at 4 °C. The mixture was IP by incubating with 50 µL Protein A beads (Sigma Code: P9424) at 4 °C for another 2 h. After washing twice with high‐salt buffer consisting of 50 mm Tris‐HCl (pH 7.4), 1 m NaCl, 1 mm EDTA, 1% NP‐40 (v/v), and 0.1% SDS (w/v) and twice with IP buffer, bound mRNAs were eluted from the beads by incubating with 6.7 mm N^6^‐methyladenosine (Sigma Code: M2780) in IP buffer and recovered via phenol‐chloroform extraction and ethanol precipitation. Then, 50 ng of the IP mRNA or pre‐IP mRNA (input control) was used for library construction with NEBNext ultra RNA library prep kit for Illumina (NEB, Beijing, China. Code: E7530). High‐throughput sequencing was performed on the Illumina HiSeq X sequencer with a paired‐end read length of 150 bp according to the standard protocol (Zhou *et al*., 2021). The sequencing was carried out with two independent biological replicates, and each RNA sample was prepared from a mix of at least 10 apple leaves.

The m^6^A‐seq data were analysed as previously described by Zhou *et al*. (2019). Here, the apple GDDH13 (https://iris.angers.inra.fr/gddh13/the‐apple‐genome‐downloads.html) was used as the reference genome. The m^6^A peaks were visualized using Integrated Genome Viewer (IGV, 2.8.0; http://www.igv.org).

### RNA‐seq

The input reads of the m^6^A‐seq were used for RNA‐seq analysis as previously described (Trapnell *et al*., 2010). The uniquely mapped reads of each sample were assembled by Cufflinks. Gene expression level was calculated as fragments per kilobase of exon per million mapped fragments (FPKM) using Cuffdiff, which provides statistical routines for assessing the differential gene expression levels (Trapnell *et al*., 2010). Differentially expressed genes were defined based on a cut‐off criterion of FPKM fold change ≥2 and *P* < 0.05.

### GO and KEGG analyses

The clean reads obtained after removing the adapter and low‐quality reads were aligned to the apple reference genome GDDH13 by HISAT2 (Kim and Langmead, 2015). The featureCounts (Liao and Smyth, 2014) was used to count the reads mapped to each gene. The DEGs of Ribo‐seq and RIP‐seq were further subjected to GO analysis on the agriGO database (version 2.0; http://systemsbiology.cau.edu.cn/agriGOv2/). Meanwhile, the DEGs of m^6^A‐seq were subjected to KEGG (http://www.genome.jp/kegg/) analysis.

### mRNA stability assay

The mRNA stability was assessed as previously described (Duan *et al*., 2017) with minor modification. Briefly, tissue‐cultured WT and *3* *5S::MhYTP2* line OE‐2 plants were grown on modified MS agar with 0.2 mm actinomycin D (transcription inhibitor), as described in the section on WT and *35S::MhYTP2* plant treatment. The tissues were collected 0, 5, 15 and 20 days after adding transcription inhibitor and immediately frozen in liquid nitrogen. The tissues were stored at −80 °C until further gene expression analysis.

### Western blot analysis

The proteins were separated by 10% SDS‐PAGE and transferred to an Immobilon‐P PVDF membrane for western blotting. The membrane was blocked with 5% non‐fat milk in PBST buffer for 2 h at room temperature. The immunoblotting was conducted by incubating with anti‐GDH1L antibody (1 : 10 000) at room temperature for 2 h, followed by incubation with HRP‐conjugated anti‐rabbit IgG secondary antibody (1 : 10 000) at room temperature for another 2 h. The immunoreactive bands were visualized using the enhanced chemiluminescence detection kit, as mentioned earlier. The antibodies were synthesized from the Wuhan Institute of Biotechnology (China) (www.atagenix.com).

### Statistical analysis

All data were analysed using IBM SPSS Statistics 21 (IBM Corp., Chicago, IL) and graphed with Sigma Plot 12.0 software (Systat Software, CA). Data are presented as the mean ± standard error (SE) of the mean of triplicates for each measurement. Data were analysed using an independent *t* test (*P* < 0.05) or subjected to one‐way analysis of variance (ANOVA).

## Conflicts of interest

The authors declare no competing interests.

## Author contributions

GTL and LCH conceived the project, designed and implemented the experiments, and wrote the paper. MFX, HL, FXM, YZH, WN, JQ and ZXZ implemented and assisted the experiments. LCH and MFW conceived the project, designed the experiments, discussed the results and edited the paper.

## Supporting information


**Appendix S1** m^6^A domain analysis of mRNA sequences.
**Appendix S2** Digoxin labelled RNA oligonucleotides of *MdMLO19*, *MdMLO19‐X1* and *MdGDH1L* for EMSA.
**Figure S1** MhYTP2 conserved motifs analysis of YTH‐domain family proteins in *Arabidopsis* and human.
**Figure S2** Confirmation of transgenic *35S::* *MhYTP2*‐*Flag* apple callus lines MhYTP2‐F1 and MhYTP2‐F2.
**Figure S3** Changes in APX and POD enzyme activities in apple leaves under normal and infected conditions.
**Table S1** The amino acid sequence alignment of MdMLO19‐X1 identified by us and MdMLO family reported in the literature (Pessina *et al*., 2014).
**Table S2** Related comment information of transcription elongation factors (MD17G1038800, MD14G1104300) and translation initiation factors (MD10G1126600).
**Table S3** Sequences of primers for quantitative real‐time PCR.

**Table S4** Differential m^6^A peaks and differentially expressed genes between the *35S::MhYTP2* line OE‐2 and the WT plants.

**Table S5** m^6^A‐containing transcripts identified via m^6^A‐seq in the WT plants.

**Table S6** m^6^A‐containing transcripts identified via m^6^A‐seq in the *35S::MhYTP2* line OE‐2.

**Table S7** Target genes of MhYTP2 identified by RIP‐seq.

**Table S8** Genes with differential translation efficiency and expression levels between the *35S::MhYTP2* line OE‐2 and the WT plants.
