# Supplementary material for: The m6A reader MhYTP2 regulates MdMLO19 mRNA stability and antioxidant genes translation efficiency conferring powdery mildew resistance in apple
Source: Plant Biotechnol J. 2021 Nov 17;20(3):511–25. doi: 10.1111/pbi.13733 (PMC8882777; doi:10.1111/pbi.13733)
Supplement: Supplementary file 1 — Appendix S1 m6A domain analysis of mRNA sequences. Appendix S2 Digoxin labelled RNA oligonucleotides of MdMLO19, MdMLO19‐X1 and MdGDH1L for EMSA. Figure S1 MhYTP2 conserved motifs analysis of YTH‐domain family proteins in Arabidopsis and human. Figure S2 Confirmation of transgenic 35S:: MhYTP2‐Flag apple callus lines MhYTP2‐F1 and MhYTP2‐F2. Figure S3 Changes in APX and POD enzyme activities in apple leaves under normal and infected conditions. Table S1 The amino acid sequence alignment of MdMLO19‐X1 identified by us and MdMLO family reported in the literature (Pessina et al., 2014). Table S2 Related comment information of transcription elongation factors (MD17G1038800, MD14G1104300) and translation initiation factors (MD10G1126600). Table S3 Sequences of primers for quantitative real‐time PCR. [file PBI-20-511-s005.pdf]

Supplementary Dataset

Appendix S1 m^{6}A domain analysis of mRNA sequences.

MdMLO19-mRNA

UAUAUAUAUACUUAUCUAUUUGCUUACUAGCUAGCUAGCUAAUUAAAAGUUCAUA

AAUACCAUUAUUAACUUCUUAGUGCAAUAGCAUUAUUUGGUAGUGAGGAAAUUAG

GGUUUCAAUUAAAGCAUGGCAGGAGGAAAAAAAGGAAGAUCUUUGGAGCAAACAC

CAACUUGGGCAGUUGCCGUUGUUUGUUUCGUUUUGGUUUUAAUUUCAAUCCUCA

UUGAAUAUUUCAUUCAUUUAAUUGGAAAGUGGUUGAAAAAGAGAAACAAAAGAGC

UCUCUAUGAAGCACUUGAGAAGAUCAAGUCGGAGCUUAUGCUAUUAGGGUUCCU

AUCCUUGCUUCUAACAGUAGGACAAGGCCCCAUUUCAAAUAUUUGUAUAUCGAAG

GCUGUUGGAGCAACUUGGCAUCCAUGCAGUAAGAAGCAAGAGGUCAAAUCAGACA

AGAACGAAGAUAAGAGUAGUGUUUCAGAUGACAACGCCCGCAGAAGGCUUCUCUC

AGCCUUGGAUUCCAGUGGGGGUGGCCGACGUGUUUUAGCAGCUGCUGGAUAUG

ACAAAUGUGCUGCCAAGAACAAAGUUCCAUUUGUGUCAUAUUAUGGGAUCCACCA

GCUUCACAUACUCAUCUUUGUGCUAGCUGUCUUCCACGUGCUUUACUGCAUAAC

AACCCUAGUUUUGGGCAGAGCAAAGAUGAGAAAAUGGAAAACAUGGGAACUGGAA

ACAAAAACAGCUGCCUACCAAUUCUCACAUGACCCGGAAAGAUUCAGAUUUGCCA

GAGACACAUCCUUUGGGAGAAGACACUUAAGCUUUUGGAGCCGUUCUCCCAUUA

GCUUAUGGAUUGUUUGCUUCUUCAGACAGUUUGUAAGAUCUGUGCCCAAAGUUG

AUUACUUGACUCUGCGACAUGGGUUUAUUGCAGCACAUUUGGCUCCUCAAAGUC

AAACUAAGUUUGACUUCCAGAAGUACAUUAACAGGUCACUUGAAGAGGAUUUCAA

GGUUGUCGUAGGGAUCAGCCCAACUAUUUGGUUAUUUGCUGUAUUAAUCCUCAU

GUCAAACACCCACGGCUCACGCUCUUAUCUGUGGCUCCCAUUUGUUCCCCUAGU

UAUGAUCUUAAUGGUAGGGACAAAGCUGCAAGUUAUAAUAACAAAGAUGGGGCUC

AAAUUAUCAGAAAGAGGUGAAGUGGUUAGGGGAACCCCACUGGUUGAGCCUGGU

GACCAUCUCUUCUGGUUCAACAACCCCCGACUCCUGCUUUAUAUCAUCCACUUUG

UUCUCUUUCAGAAUGCUUUUGCACUUGCUUUCUUUGCAUGGACUUGGUAUGAAU

UUGGCUUGAAGUCUUGCUUCCAUGAGAAGUUGGAAGAUGUCGUUUUAAGAAUAU

CAAUGGGGGUGAUCAUACAAAUUCUGUGCAGCUAUGUAACUCUUCCUCUUUAUG

CAUUGGUAACACAGAUGGGUUCAACUAUGAAGCCUGUAAUCUUCAACGACAGAGU

GGCGACUGCACUCAAAAAAUGGCACAUCGCAGCGAAGAAGCACGUAAAACACAAG

AAUGCAAGUCCAGCCAGUGCACCAGGCACUCCUUUGCACUCCAUGUCCCCUGUU

CAUCUACUACGCAACUAUAAAUAUGAACAAGACAUUGACAGCAUUCAGACAUCACC

AAGAAUGCCUUAUUUUGAUAACGAAGGUUCAGACUCACCGUUUCAUCACCAAGAC

AAUUUGACUUGGUCUCAGCAAGGUACAAACAUGGAGGGUCAGAAGGAGGAGAUU

AGUGCUCAUGGACCUAACGCAGAGAGCAAUGCUUUAGGCGCUUAUGGUUCGAUA

AUUCAACAUGAGAUUCAAAUUCACUCGGCGGCACUCACAUUUGAGAAAACAGAAA

GAAGCUGAGAGAUUAGGAAGUAAAUGGCAUACAACUUGCAACAUUACAAUCAAAC

CUAAAGCCUGAUCUCAUAAGCUUCCCAAUGUCACUGUACAUGAAGACACUCGGUG

CUAGACAUCAUGAGUUGUACAACUGUAACGUAAAUUUUGAUAUUCAUGCAAAUAA

ACCUUCACCUUGAUCACGUUGAGGCAGUGUAGAUAUCUCCAUAUGUAAUCACUUU

CCCGGCAACACAUUGAUGUGUUAAUAUACACACUGUGAGGAACA

MdMLO19-X1-mRNA

UCUCUUUCUCUCUCUCUCUCUCUCUCAUAUUCAUGAUUCGAUAACUUCAGUUGUUUC

AUUCAUUGUUCCUUGUCAAUUGGGCAACUUAUUGGAAACUUCAGUUGUUUCAUU

UCAUUGUUCUUUGUCAAUUGGGUUCUUUGUCAAUUGGGCAACUUAUUCGAAACU

UAAGUUAUUUCAUUUCACUGUUCUUUGUCAAUUGGGCAACUUAAUCCAGAAAACA

UGGCAGCGGCAACGUCUGGAAGAUCGUUAGAGCAAACGCCGACGUGGGCUGUUG

CCGUAGUCUGUUUUGUUUUGGUCUUGAUUUCAAUAAUCAUUGAACACAUAAUCGA

ACUUAUAGCAAAGUGGUUAAAGAAGAAACACAAAAGCGCCCUUUACGAAGCACUA

GAAAAGAUCAAAUCAGAGCUUAUGUUAUUGGGGUUCAUAUCCUUGCUCUUAACUG

UGGGACAAGGACCAAUAUCAAACAUAUGCAUAUCACAGAAAAUUGGCAACACUUG

GCAUCCGUGUGGCAAGAAGCAAGAAGACAAAUUGAAUAAGGAAGAGGAUUUGGAG

UAUGUGGAUGAAACCCCGAGCAGGAGACUGCUCUCUCUGUUUAACGAGACGGGC

GGUGGCUCACGCCGUGUUCUGGCUGCUGCUGGCACCGACAAAUGUUCAGCAACG

GGUAAAGUACCUUUUAUAUCUGCGGAUGGUAUUCAUCAACUGCACAUUUUUAUCU

UCGUGUUGGCUGUUUUCCAUGUCCUUUAUUGCAUCCUCACAAUGGCUUUAGGAA

GAGCCAAGAUGGGAAGUUGGAAGCGGUGGGAAAAGGAAACAAGAACAGUUGAAU

AUCAAUUUUCUCAUGACCCAGAGAGAUUCAGGUUUGCAAGGGACACAUCUUUUG

GGAGGAGGCAUAUGAGUUUCUGGACCAAAACACCUUUCCUUAUGUGGAUAGUUU

GUUUUUUUAGACAAUUUGUAAGGUCCGUUCCCAAAGUGGAUUACUUAACCUUGC

GGCAUGGGUUUAUCAUGGCACAUUUGGCACCCCAAAGCCAUCAGAAUUUCAAUUU

CCAAAAAUACAUAAACAGAUCACUUGAAGAGGAUUUCAAGGUAGUUGUGGGAAUC

AGCCCUCCAAUUUGGUUCUUUGCGGUGAUAUUCAUACUUUUUAACACCCACGGC

UGGUAUACUUACUAUUGGCUACCGUUCAUCCCAUUGAUUAUCAUCCUCUUGGUG

GGGACCAAGCUACAGGUGAUCAUCACGAAAAUGGGUCUUCGAAUUCAAGAAAGCG

GAGAGGUUGUAAAGGGGCUUCCUGUGGUUCAACCUGGUGAUCAUCUCUUUUGGC

UCAACCGCCCUCGUCUCAUUCUCUACCUCAUCAACUUUGUUCUCUUUCAGAAUGC

CUUCCAGCUUGCUUUCUUUGCAUGGAGUUGGUAUGAAUUUAGCUUGAAAUCUUG

UUUUCACGAGCACACUGAGGACGUGGUCAUCAGAGUUUCAAUGGGGAUCCUCAU

ACAGAUUCUCUGCAGUUAUGUCACUCUACCCCUCUAUGCCCUUGUCACACAGAUG

GGCUCAACCAUGAAACCAACCAUAUUCAAUGAAAGAGUAGCCGCAGCUUUACGCA

ACUGGCAUCACACAGCCAGGAAGCACAUAAAGCAGAACAAAGGCUCCGUAACCCC

AAUGUCUAGCAGACCAGUCACUCCAUCCCACCACACGUCCCCCGUCCACCUCCUU

CGGAACUAUCGGAACGAAGUGGAUAGUUUCCAUGCAUCACCAAGAAGAUCAAAUU

UCGAGGGUGAACGUUGGGCCAACGAAUCACCCUCCCCCUCACACCACCUCCAUG

UAGAUGGCAGUUCAUCCUCUUACCACCACCAUAUUGAAAUGGGAGAUGUAGACCG

UGAGAGGGUUGAUGUCAAUGAACAAAAUUCGGUUGAUGGGAAAACUACAAUAACU

GAUACUAGUAUUACUGUCAUGGAACCUGCUCAAACACAACACGAAAUUAACAUGG

AGCGGUCGAAAGACUUCUCAUUUGACAAGAGGCAACCUACACAAUAGCUCGAAAA

UUUGUUGCAUUAUGCAUUAGAGAUCGGCAAUGAUGGUGACAAUGAUGACAGCAA

GGACGAUGACGCAAUGAUCGGAGAGAAUGGAGAUGCUUGAAGCCACACAUUUUG

CAUCCUGCCGAGUAGUGUUUUUUUUUUUAAUAUAUAUUUUUUACUCAUUUAUGUU

CUUGAUUUGCUUCAUAUUUGGCAGGGAUGGAGAUACACAUGCAGUGACUGUCUC

AAUUGUUUUACUGUGCCUUUAUGUUAUAGCAUUUUUUCUGUAAAUAAAUGCCUCU

AAAUCUUAAGCACAAAGUCACGAUCCUGAAUGCCAAAGAGCAUUAAUAUUCUGUU

UGAAGAGUUGAUAAUUGCCUCUCACAAUAUAUAUGCAGUUGAUGAC

MdGDH1L-mRNA

GGGUUGGCGACUUCGAUCAUGCGUCCUACAGUCUGCAUCGAUCUCUUCCAUCUC

UGCUUCGUGAUCCCUUCGAUCCCUGCUUCCUCAUCUUUUCGAUCUCUGCUUCAC

GAUCUUCUCGCCCCCUCAUCCUGCACCUAAUUUCUCUAGCCUUCAAGAGGCAAUC

AUGGUUAGAUUUCAUGCGGUUCAUGAAAGACUCGGUCAGGCUUUAGAAGGUUUG

CUUUAUGAGCUUUGCCCAUCAGAUGAAGUUGAGGAACAAACAGAUCUUUUAUCAA

UUCAAACUAUUCUCGCUUUGCUGCACACGUUUGAUAUAAGUCCGUUUGGGUUUG

ACGAGAUUCAGAGUACUUCUUAUCAAAUUGGUCGCACGGACCACUACAAGGAAGA

CAUCUUCCAAACCCUCUACAUAGCAUCGAGAUUAUUGCCCCUUCGAUUCCUCUCC

CACUGCCUCAAGCCAAAACCAAACCCAGCUCCAACCUCCAGUCCUCUACAAAUCA

AACCUCACAAAAAUCCGAUAUUGGAGGUGAUAAGUAGCUGGUUUUAGCUUCUGAG

GCAGACACUUGAAGGAGCAAAGAUGAAUCCAUUAGUGGCAACGAACAUAAACUUU

AAGUUUGCUGCUUGGCUUUUGGGAUUGGACUUCAAGCUUGAGAAAAGUUUACUU

AUACCAUUUAGGGAAAUCAAGGUUGAGUGUACUAUGCCAAAAGACGAUGGCAGUU

UGGCUUCAUUUAUUGGCUUCAGGGUUCAACAUGACAAUGCUAGAGGCCCCAUGA

AGGGAGGAAUCAGAUAUCACCCAGGGCUGAUGACAUGGAAGACGGCGGUAGUCA

ACAUCCCAUACGGGGGUGCCAAAGGGGGUAUAGGAUGUAAUCCAGGGGAGUUGA

GUCUUUCCAAACUAGAGCAACUCACCAGAGUUUUCACACAAAAGAUGCACGAUCU

UAUCGGAAUCCACACCGAUGUUCCAGCACCAGAUAUGGGGAUAAGUCCACAGACC

AUGGCAUGGAUACUAGACGAAUACUCAAAGUUUCAUGGCUACUCACCUGCAGUAG

UGACUGGAAAACCUAUUGAACCCUGAUUAUGGGAGGAAGAAUACGCUUCAUUGCC

AUCUUUAGAUCAAAACAAAGUGAAAGCUUUUGACUGAAUUUUGCAACCGCUACUU

UGCUGCUUUGGAAUCCUGAUUCUGGGAGGAACAAUACGCUUCAUUGCCUUCUUU

AGAUCAAGAUCUUAUUGCAACGUUGUGAUCUGCUCUUCUUGCUGCCUUUAUCUA

GGAAUCCGCACCUAUUGCAUUUGAAUCAAGGCUUUGGUAUGUCACCUAAACUAAU

AUACAUUGUUUCCCUGUGCUUUCCGUUUGGAGUUAAUGCAAAUUUAUCAUGUUU

CAGUCGAUUGUUUGGUUUUAUCAGCUUAUGUCAUUCUUUUGCUACCUGCUUUGU

UUCUCAUUCUUGAUUAUCUCUGUAAUCUUUUGAUACUUCACAAGUUCAUUGGUUU

CUUUGUUUUUGUUGGUUUUAACUUGGUCUCAACCAUUCUAUUAUUGUUCAAAGG

UGCAUUGGUGUCAUCUGUGAUUUUGCUUUAACUAGCUGUUCCUAAUUCUUAGUC

GAAUGUUGGUUAAUAAAAUGGUGAAAGAGAUUGAUCGAAGCGAACAACAAAUUCU

AGAAGGCGAUUUGGUAAAUUUUUUUUAAGGGAACUGAUUUAAGGCUCGAAACGAU

GAAAAGAAUGAAGGAACGAUAGUGAAAUAUUUCGAUACUGCCCCAGGACCCAGUA

CAAAGAAAUCAGUGAAAAUAGAAAAAACAAGGUUCAGUUUGCGUGUUUAUGAUUG

AAAAUUUCGAUUUAUUUACGAAAUAACCACCCUUCAGCGUGCACGUGUCGAGCAA

AAGCAAACCUGACUUUGUUCCAGAGAUCUCUUCGAUGACAAACCCAUUUUGCUCC

AGAGCUUUUCAACGACAAAGUCGAUUCGGCGCCUCACCGUCGCACACCUCGCCU

UCCCCUCGUCGUCGCACACCUCACCUUACCUCGCCGCCGCAGUCCUUACCAGAA

CUCGAAUCAAUGCCAAAACUCACGGAUUAGGCUGAGAUUGAGAUAUAUGGGAGGA

UUUGUGGACGGAUUUCAGGGAGAGAGGCCCACGAGAACGACACAGACGGUCUCG

CCUCCAUCCUAUCCUCCUUUCGCCGCCGUCUACCUUCCCUCGCCUAUGUCGUCU

UACUCCACCGUUUGCGUCAGGAAGGCCCGUGCGUGGUUUUUGGGUGGGGAUU

UCAAAUUUCAAGGGGGAGAGAGGCUGUGGUUCGUUUCGAUCGGGCCGAGCCUCU

AUGAUUCCAUUGGAUCGAGGUGAUGGGGACAAGGCGAGAGGUUGAGGGAAGCCG

GCUCUAAGACUCUGCAUCUCUCACUUUCUCAUCUCUCAAACCACUUCUCUCUCUC

CCAUCUCUCCCACUGUCUCCGUCUCUUGGUUUUCUCGCUCACUCUCUGUCCACU

GAAAUUUCAAGAAAUUCUGAGAUGGAAAAGGUUACGGACAAAUUGGAAGGUAGAA

AUUCCAAUAUGAGUUCUCCAGCCGAUUUGAGGUGGAGUUUUCUAUUAGGGAAAC

GUUAGGACCUGAUUGUUCCACAUCGAAAAAACAGCAGCAGUGGGUCCUAUUUAAA

AUUAAUCUAAUUGAAGAGACUAAGGACUGGGCACCUUCUACAAGCUGCAGUACUA

CUAUUUGAGGUUUUGCAUCAUUGGAACAUCAGGCAUAGGUCAUUGCUUUAUCUA

AGGAGAUGGCAAAAUCUUGGAUUGCCAAUUGAGGAUAUAGCUGCAAUUCAAAUUC

AGACUGCUUUCCUUGCACAUGUUGCUCAUGGAAUACUCUUCCCAAACUUAAUCAG

UAAGCGAGUGAACGACAUAAGUUUCUAAAUUACAGAGAGGAGAUAUCUCAUUACA

AGGAACUCAAUCCAACAGAACUCAGUCCAAAAGAACAUUUGCUACUCUUAAAAGCA

UUAUGUGGACUCCAAGCUCAAUGCCCAUUUCCACCCAAAUUGAAUCUCAAGUUCU

UCUGCUUUCGAAGACGGCAUGAUUUGACAAGAUCAUCAACAAGGAAAUACCGAAU

AAGGUGGUUUUGAAGACGAUAAGGUCUUGAAGAAGCAGCUGACGCUGAUUUAUC

CGAGAGACGAAGGCAAGCCUCUACUAAACAGCCCAUAUUGGCCGACGACAGAGAU

UUAGUAUGCAAGGUCGGCAAGCUGGUGAGGUUUCCAAGGACGUCACCAAUUUCG

UGAAGUUGCUGGAGAACAAAUCGGAAAAGUUCACUGCUUUCUUUGUGGAGAAGG

GGUAGUACGUUAGUCGAUGGAAGGUAUUAUUUCUUGAUUUUCUUUAAAAAAUUAU

CGAAGAUUACAAUUGAGAUUCUAGAAUUAUGAGAAUUUGUUUAGGAAUUGAGAGA

UUGACUUGCUGCUUGCGAAUUCAGAAAAUUUAUGGUUUUUUAUAUGAGAAUUUU

GGUUUAGGAUAUCGAAAGAUUGUCUUUGAUGUAUGAUAUUGAAUUUGGAUGAAA

UUUAUUAGAUUUUGCCUUUUUUUUAGGCCGUUGUAUGUAUUAAGUUUGUAAUUA

GGAACUGUUUUGUAUGAUUUUCUGUAUUCGUAAUUGUUCAGCAUAAUAAAAUAUA

GGGUUUUUUC

Here the highlighted ones represent the common m^6A domains.

Appendix S2 Digoxin labeled RNA oligo-nucleotides of *MdMLO19*, *MdMLO19-X1* and *MdGDH1L* for EMSA.

1) UAUAA(MdMLO19)

UAGGGACAAAGCUGCAAGUUAUXAUAACAAAGAUGGGGCUCA

2) UAUAA(MdMLO19-X1)

CAACGGGUAAAGUACCUUUUAUXUCUGCGGAUGGUAUUCAUC

3) UGUAA(MdGDH1L)

UUUUAGCUGGAAGUGAUUAUGUXAAAGCAGCUGAAAAGGUGC

Here X indicates either an A or an A with m^6A modification.

Figure S1 MhYTP2 conserved motifs analysis of YTH domain family proteins in *Arabidopsis* and human.

YTH domain

```
AtECT5      NADFVTDYTNAKLFIIKSYSEDNVHKSIKYNVWASTPNGNKKLDAAYREAK----DEKEP
AtECT9      QQELLSQFRDAKFFVIKSYSEDNVHKSIKHCVWASTKNGNKKLDAAYREAK----KKDVA
AtECT10     HPEFVTDYKNAKFFIVKSFSEDNVHRSIKYNVWASTPHGNKKLDTAYRDAE----KMGGK
AtECT1      GENFPESFVKAKFFVIKSYSEDDVHNCIKYGAWSSTPTGNKKLNAAYYEAK----ENSQE
AtECT3      KIDFPETYTEAKFYVIKSYSEDDIHKSIKYSVWSSTPNGNKKLDASYNEAK----QKSDG
AtECT2      KEDFPVDYANAMFFIIKSYSEDDVHKSIKYNVWASTPNGNKKLAAAYQEAQ----QKAGG
AtECT4      RDDFPVEYKDAKFFIIKSYSEDDVHKSIKYNVWASTPNGNKKLDAAYQEAQ----QKSSG
MhYTP2      GTDIYDHYADAKFFVIKSYSEDDVHKCVKYNVWASTPNGNNKLHAAYQEAQ----EKSGG
AtECT6      KEDFSIEYSDARFFVIKSYSEDDVHKSIKYGVWSSTLNGNKKLQSVYEDAQRIATEKSRE
AtECT7      KEDLRIDYSNAKFFVIKSYSEDDVHKSIKYNVWSSTLHGNKKLQSAYEDAQRIATEKSCE
AtECT8      LPSFQTKYEEAIFFVIKSYSEDDIHKSIKYNVWSSTLNGNKKLDSAYQESQKKAADKSGK
AtECT11     LPDFQTDYEDAKFFVIKSYSEDDVHKSIKYSVWSSTINGNKKLDAAFRDAETKTLEDGKK
HsYTHDF2    PKDFDWNLKHGRVFIIKSYSEDDIHRSIKYNIWCSTEHGNKRLDAAYRSMN-------GK
HsYTHDF3    PKDFDWNLKNGRVFIIKSYSEDDIHRSIKYSIWCSTEHGNKRLDAAYRSLN-------GK
HsYTHDF1    PKEFEWNLKSGRVFIIKSYSEDDIHRSIKYSIWCSTEHGNKRLDSAFRCMS-------SK
AtCPSF      RTSHPLPQGVNRYFVVKSNNRENFELSVQQGVWATQRSNEAKLNEAFDSVE---------
AtECT12     ANKNSKPGYRTRYFIIKSLNYDNIQVSVEKGIWATQVMNEPILEGAFHKSG---------
HsYTHDC1    TSKLKYVLQDARFFLIKSNNHENVSLAKAKGVWSTLPVNEKKLNLAFRSAR---------
HsYTHDC2    KSPSPRPNMPVRYFIMKSSNLRNLEISQQKGIWSTTPSNERKLNRAFWESS---------
                         :::** .  :.  .     *.:   .:  *   :
```

YTH domain

```
AtECT5      CPLFLLFSVNASSQFCGVAEMVGPVDFEKSVDYWQ------QDKWSGQFPVKWHIIKDVP
AtECT9      CPVFLLFSVNASSQFCGVAEMVGPVDFNTSVEYWQ------QDRWSGHFPVQWLIVKDVP
AtECT10     CPIFLFFSVNASGQFCGVSEMVGPVDFEKDAGYWQ------QDRWSGQFPVKWHIVKDIP
AtECT1      CPVYLLFSVNASGQFVGLAEMVGPVDFNKTMEYWQ------QDKWIGCFPVKWHIIKDIP
AtECT3      CPVFLLFSVNTSGQFVGLAEMVGPVDFNKTVEYWQ------QDKWIGCFPVKWHFVKDIP
AtECT2      CPIFLFFSVNASGQFVGLAEMTGPVDFNTNVEYWQ------QDKWTGSFPLKWHIVKDVP
AtECT4      CPVFLFFSVNASGQFIGLAEMKGPVDFNKNIEYWQ------QDKWTGSFPLKWHILKDVP
MhYTP2      CPVFLFFSVNASGQFVGIAEMVGLVDFNKNVEHWQ------QDKWTGCFPVKWHIVKDVP
AtECT6      CPIFLFFSVNSSGLFCGVAEMTGPVSFDRDMDFWQ------QDKWSGSFPVKWHIIKDVP
AtECT7      CPIFLFFSVNASGLFCGMAEMTGPVSFDKDMDFWQ------QDKWSGSFPVKWHIIKDVP
AtECT8      CPVFLFFSVNASGQFCGVAEMIGRVDYEKSMEFWQ------QDKWTGYFPVKWHIIKDVP
AtECT11     RPIFLFFSVNASRQFVGLAEMVGYVDFNKDLDFWQ------VDKWSGFFPVEWHVVKDIP
HsYTHDF2    GPVYLLFSVNGSGHFCGVAEMKSAVDYNTCAGVWS------QDKWKGRFDVRWIFVKDVP
HsYTHDF3    GPLYLLFSVNGSGHFCGVAEMKSVVDYNAYAGVWS------QDKWKGKFEVKWIFVKDVP
HsYTHDF1    GPVYLLFSVNGSGHFCGVAEMKSPVDYGTSAGVWS------QDKWKGKFDVQWIFVKDVP
AtCPSF      -NVILIFSVNRTRHFQGCAKMTSRIGGYIGGGNW--KHEHGTAQYGRNFSVKWLKLCELS
AtECT12     -RVILIFSVNMSGFFQGYAEMLSPVGWRRDQIWS--QGGGKNNPWGRSFKVKWLRLSELP
HsYTHDC1    -SVILIFSVRESGKFQGFARLSSESHHGGSPIHWVLPAGMSAKMLGGVFKIDWICRRELP
HsYTHDC2    -IVYLVFSVQGSGHFQGFSRMSSEIGREKS-------QDWGSAGLGGVFKVEWIRKESLP
              : *.***. :  * * :.: .                         * : *    .:.
```

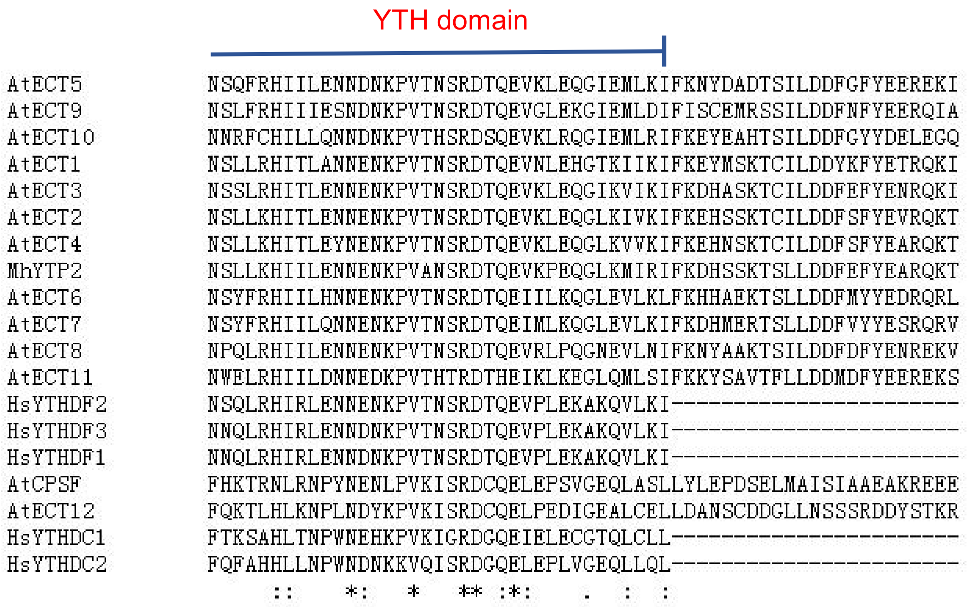

The asterisk represents the same amino acid conserved among these proteins. The dot represents the level of the similarity of amino acids conserved among these proteins. The level of the similarity indicated by two dots is higher than that of one dot.

Figure S2 Confirmation of transgenic *35S::MhYTP2-Flag* apple callus lines MhYTP2-F1 and MhYTP2-F2.

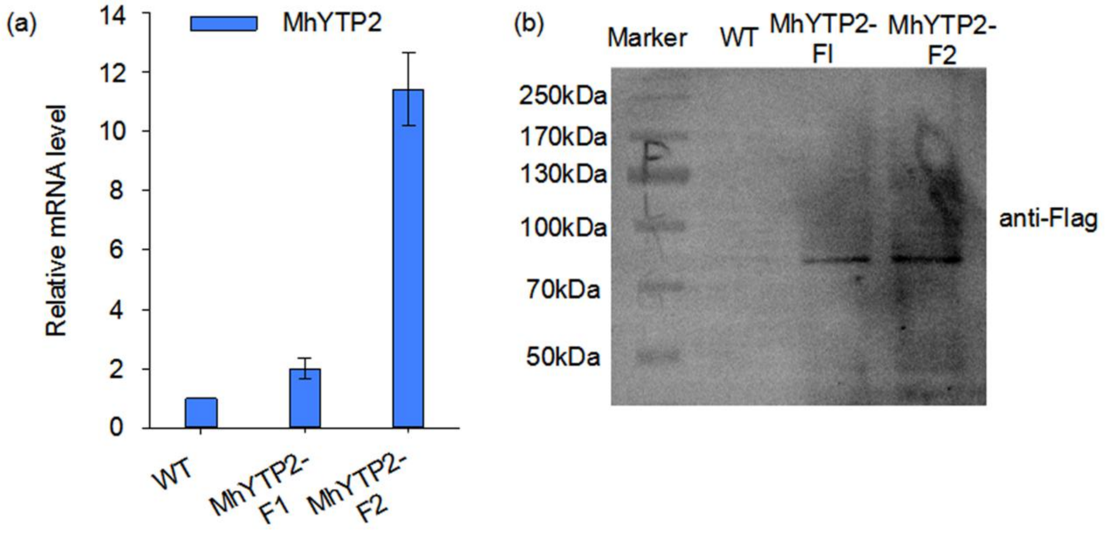

Confirmation of transgenic *35S::MhYTP2-Flag* apple callus lines. **a** Quantitative RT-PCR analysis of *MhYTP2* expression in apple callus of WT, MhYTP2-FI and MhYTP2-F2. **b** Detection of MhYTP2 accumulation in apple callus by western blot.

Figure S3 Changes in APX and POD enzyme activities in apple leaves under normal and infected conditions.

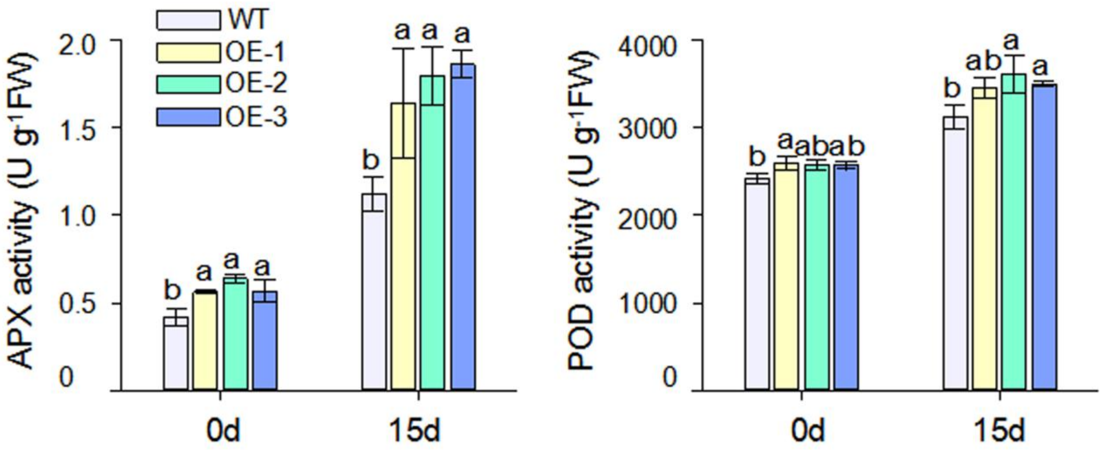

Table S1 The amino acid sequence alignment of MdMLO19-X1 identified by us and MdMLO family reported in the literature (Pessina *et al.*, 2014).

Gene	Accession number	Amino acid sequence similarity（%）
MdMLO1	MDP0000177099	28.90
MdMLO2	MDP0000240125	19.33
MdMLO3	MDP0000168575	22.99
MdMLO4	MDP0000207002	37.84
MdMLO5	MDP0000163089	59.05
MdMLO6	MDP0000119433	34.70
MdMLO7	MDP0000123907	58.74
MdMLO8	MDP0000218520	15.55
MdMLO9	MDP0000320797	31.13
MdMLO10	MDP0000196373	32.24
MdMLO11	MDP0000239643	60.63
MdMLO12	MDP0000133162	38.98
MdMLO13	MDP0000142608	20.44
MdMLO14	MDP0000191469	21.25
MdMLO15	MDP0000141595	32.33
MdMLO16	MDP0000191848	37.50
MdMLO17	MDP0000145097	29.95
MdMLO18	MDP0000928368	42.83
MdMLO19	MDP0000168714	63.01
MdMLO20	MDP0000134649	37.38
MdMLO21	MDP0000133760	38.30

Table S2 Related comment information of transcription elongation factors (MD17G1038800, MD14G1104300) and translation initiation factor (MD10G1126600).

**Gene name**	**Description**	**GO**
MD17G1038800	PREDICTED: uncharacterized protein LOC103404360 [Malus domestica]	GO:0006368(transcription elongation from RNA polymerase II promoter);GO:0070449(elongin complex)
MD14G1104300	PREDICTED: uncharacterized protein LOC103954799 [Pyrus x bretschneideri]	GO:0003677(DNA binding);GO:0003682(chromatin binding);GO:0005634(nucleus);GO:0006351(transcription, DNA-templated);GO:0008270(zinc ion binding)
MD10G1126600	PREDICTED: uncharacterized protein LOC103946617 isoform X1 [Pyrus x bretschneideri]	GO:0003743(translation initiation factor activity);GO:0005737(cytoplasm);GO:0006413(translational initiation)

Table S3 Sequences of primers for quantitative real-time PCR.

Gene	Primer	Sequence (5′–3′)
MdMTA	RT-*MdMTA*-S	GAGAACGGTGGTGTGGAGTT
	RT-*MdMTA*-A	TCACCACTTGCTGCTGATTC
MdMTB	RT-*MdMTB*-S	TCAAGGTGGTTGGTTTCCTC
	RT-*MdMTB*-A	GTGAAGCAGAATGAGCCACA
MdFIP37	RT-*MdFIP37*-S	ATGCAGGCAAGAAGATTGCT
	RT-*MdFIP37*-A	TGCCAATCTCCTCATTTTCC
MdALKBH2	RT-*MdALKBH2*-S	TTCCCCAATTGGTTTACAGC
	RT-*MdALKBH2*-A	TCAGAATGCCAGCCAACATA
MdALKBH6	RT-*MdALKBH6*-S	AGACGAGTGAGCGCTGAAAT
	RT-*MdALKBH6*-A	CGTAATGAAGTCCGGGATGT
MdALKBH9B	RT-*MdALKBH9B*-S	TCTAGGGAGCAGAGGGAACA
	RT-*MdALKBH9B*-A	TTGCCCCTAATCCACTTTTG
MdMLO19	RT-*MdMLO19*-S	GCAGCTGCTGGATATGACAA
	RT-*MdMLO19*-A	TCTTTGCTCTGCCCAAAACT
MdMLO19-X1	RT-*MdMLO19-X1*-S	TTCACGAGCACACTGAGGAC
	RT-*MdMLO19-X1*-A	TACGGAGCCTTTGTTCTGCT
MdActin	RT-*MdActin*-S	TGACCGAATGAGCAAGGAAATTACT
	RT-*MdActin*-A	TACTCAGCTTTGGCAATCCACATC
